# A Halomanganates(II) with P,P’-Diprotonated Bis(2-Diphenylphosphinophenyl)ether: Wavelength-Excitation Dependence of the Quantum Yield and Role of the Non-Covalent Interactions

**DOI:** 10.3390/ijms22136873

**Published:** 2021-06-26

**Authors:** Alexey S. Berezin

**Affiliations:** Nikolaev Institute of Inorganic Chemistry, Siberian Branch of Russian Academy of Sciences, Acad. Lavrentiev Ave., 3, 630090 Novosibirsk, Russia; berezin-1991@ngs.ru or berezin@niic.nsc.ru

**Keywords:** manganese(II), luminescence, EPR, non-covalent interaction, DFT

## Abstract

A [H_2_DPEphos][MnX_4_] [X = Br, Cl] tetrahalomanganates(II) with P,P’-diprotonated bis[2-(diphenylphosphino)phenyl]ether cation has been designed and investigated in photophysics and EPR terms. The complexes exhibit a green luminescence resulted from the Mn(II) *d*–*d* transitions (^4^*T*_1_→^6^*A*_1_) with the wavelength-excitation dependence of the quantum yield. The solid [H_2_DPEphos][MnBr_4_] complex exhibits a bright green phosphorescence (λ_max_ = 515 nm) with the high luminescence quantum yield depending on the excitation energy whereas the solid [H_2_DPEphos][MnCl_4_] complex exhibits a very weak phosphorescence (λ_max_ = 523 nm). The unexpected shorter luminescence lifetime for the [H_2_DPEphos][MnCl_4_] than for the [H_2_DPEphos][MnBr_4_] at 300 K can be a result of the higher non-radiative relaxation contribution. On the one hand, the non-covalent PH…X(Mn) interactions quench the manganese(II) luminescence. On the other hand, the PH…X(Mn) interactions are a pathway of the excitation transfer from [H_2_DPEphos]^2+^ to [MnX_4_]^2−^.

## 1. Introduction

The luminescent manganese(II) complexes have been intensively studied over the past few decades [1,2,3,4,5] on account of their interesting optical, thermal, and magnetic properties [6,7,8,9,10,11,12]. The luminescence of the manganese(II) complexes can be originated from the metal perturbed intraligand transition [6,7] and from the Mn(II) spin-forbidden *d*–*d* ^4^*T*_1_(**G**)→^6^*A*_1_(**S**) transition [6,7]. In the last case, the emission maximum is strongly dependent on the crystal field [5] parameters of the Mn(II) center. The emission of the Mn(II) center can vary from green to near-infrared range for the tetrahedral and octahedral coordinated Mn(II), respectively [13]. The coexisting of both Mn(II) coordination in the compounds can lead to observing the dual green-red luminescence, but such examples are still rare.

Currently, special attention is paid to the organic–inorganic tetrahalomanganates(II) compounds. The adjustable coordination geometry [13] make the nitrogen- and phosphonium-based molecules promising counterions for obtaining effectively luminescent tetrahalomanganates(II). The bright green luminescence of the tetrahalomanganates(II) [13,14,15,16,17,18] with the quantum yields varying in the wide range and reaching 100% (Table 1) make these compounds good prospects for creating light emission devices. The organic cations and [MnX_4_]^2−^ anions in the tetrahalomanganates(II) compounds interact through the non-covalent interactions. One of the types of non-covalent interactions is a hydrogen bond. The hydrogen bonds have a strong influence on the tetrahalomanganates(II) compounds defining the geometry, stability, and disordering of the [MnX_4_]^2−^ center. The hydrogen bonds can also increase or decrease the non-radiative relaxation probability of the excited states of the cations and [MnX_4_]^2−^ centers, influencing the luminescence lifetimes and quantum yields [19,20]. It should be noted that the hydrogen bonds can also play a role in the excitation transfer channel between different parts of compounds [20,21,22,23].

Herein, the synthesis and comparative investigation of the luminescent organic–inorganic tetrahalomanganates(II) compounds, [H_2_DPEphos][MnX_4_] (X = Br and Cl, DPEphos = bis[2-(diphenylphosphino)phenyl]ether), are reported. The [H_2_DPEphos][MnBr_4_] shows a bright luminescence at 515 nm with 42–60% photoluminescence quantum efficiency at room temperature depending on the excitation energy. The electron paramagnetic resonance, low-temperature photoluminescence measurements, and DFT calculations were performed to investigate the mechanisms of the excitation, radiative, and non-radiative relaxation processes. This work extends a class of organic–inorganic manganese(II) complexes with the hydrogen bond network, which plays an important role in the charge transfer processes between organic cation and inorganic anion. It is shown that the non-covalent PH…X(Mn) interactions can not only quench the luminescence but can also be a pathway of the excitation transfer from the organic “antenna” to the manganese(II) ion. It is worth noting that the work devoted to the bright green-luminescent tetrahedral manganese(II) dihalide with DPEphos oxide ligand [15] has shown the organic ligand acting as an effective UV-radiation antenna for the manganese(II) ion. This work highlights the importance of the intentional choice of the organic fragments with certain properties (especially an electronic structure and energy transfer possibilities through protons) when considering the tetrahalomanganates(II) synthesize strategy. The indirect excitation process of manganese(II) ion through the organic cation can be employed to improve the luminescence efficiency.

## 2. Experimental Part

### 2.1. Synthesis and Characterization Data for ***1*** and ***2***

#### General Procedure for the Synthesis of Complexes **1** and **2**

**Scheme 1:**
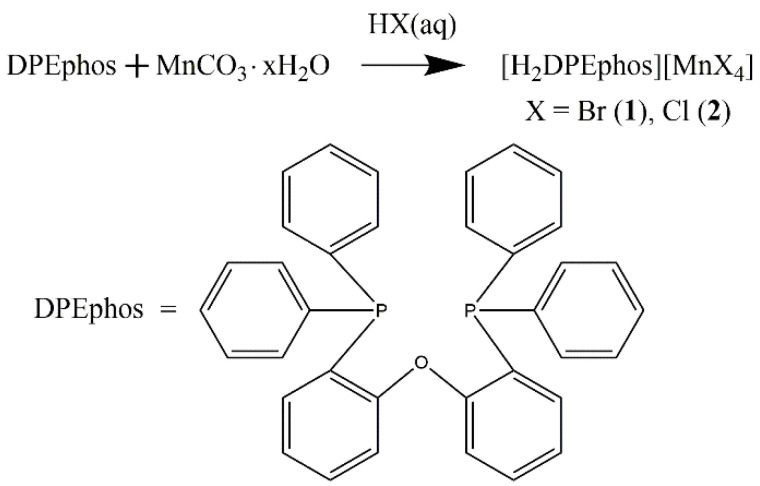
Synthesis of complexes **1** and **2**.

To a mixture of solid bis(2-diphenylphosphinophenyl)ether (DPEphos) (≥98.0%) and MnCO_3_·xH_2_O (44–46% Mn) taken in the 1:1 molar ratio, a volume of 3 mL of concentrated hydrohalic acid HX (X = Cl (37%) or Br (48%)) was added (Scheme 1). The mixture was stirred at 50 °C for 1 h. The crystals formed were precipitated from the solution. The crystals were collected through filtration and dried in the air. Using the same molar of the hydrated manganese(II) halide salts MnX_2_ (instead of manganese(II) carbonate) leads to the formation of the same products.

**[H_2_DPEphos][MnBr_4_]** (**1**) was prepared from MnCO_3_·xH_2_O (30.4 mg, 0.25 mmol) and DPEphos (134.6 mg, 0.25 mmol) using hydrobromic acid HBr (48% HBr) as solvent. Yield: 197 mg (86% Mn). Colorless prism-shaped crystals. IR spectrum is in ESI.

**[H_2_DPEphos][MnCl_4_]** (**2**) was prepared from MnCO_3_·xH_2_O (30.4 mg, 0.250 mmol) and DPEphos (134.6 mg, 0.25 mmol) using hydrochloric acid HCl (37% HCl) as solvent. Yield: 166 mg (90% Mn). Colorless prism-shaped crystals. IR spectrum is in ESI.

## 3. Methods

Suitable crystals were selected and mounted on a MITIGEN holder oil on an XtaLAB Synergy R, DW system, HyPix-Arc 150 diffractometer. The crystals were kept at a steady T = 123.01 (10) K for **1** and T = 100.01 (11) K for **2** during data collection. Data were measured using *ω* scans using CuK*_α_* radiation. The diffraction patterns were indexed and the total number of runs and images were based on the strategy calculation from the program CrysAlisPro [24]. The maximum resolutions were achieved *θ* > 73°. The unit cells were refined using CrysAlisPro [24]. Data reduction, scaling, and absorption corrections were performed using CrysAlisPro [24]. A Gaussian absorption correction was performed using CrysAlisPro [24]. Numerical absorption correction based on Gaussian integration over a multifaceted crystal model empirical absorption correction using spherical harmonics, implemented in SCALE3 ABSPACK scaling algorithm. The structure was solved with the ShelXT [25] solution program using dual methods and by using Olex2 [26] as the graphical interface. The model was refined with ShelXL [27] using full-matrix least-squares minimization on *F*^2^. All non-hydrogen atoms were refined anisotropically. Hydrogen atom positions were calculated geometrically and refined using the riding model, but some hydrogen atoms were refined freely. CCDC 2078076 and 2078077 contain the supplementary crystallographic data for this paper. These data can be obtained free of charge from The Cambridge Crystallographic Data Center at www.ccdc.cam.ac.uk/structures (accessed on 1 May 2021).

Corrected luminescence spectra were recorded on a Fluorolog 3 spectrometer (Horiba Jobin Yvon) with a cooled PC177CE-010 photon detection module equipped with an R2658 photomultiplier; with continuous 450 W and pulsed (pulse time FWHM 3 μs) 50 W Xe-lamps; with two Czerny–Turner double monochromators. Absolute values of quantum yields were recorded using the Quanta-φ device of Fluorolog 3. Temperature dependencies of luminescence were studied using Optistat DN optical cryostat (Oxford Instruments). The luminescence quantum yield at 77 K was obtained relative to the quantum yield of the same sample at 300 K [28].

EPR spectra were recorded on the Varian E-109 spectrometer in Q-band at 300 K. A 2,2-diphenyl-1-picrylhydrazyl (DPPH) standard sample was used to calibrate the magnetic field of the spectrometer. The spectra were simulated in MATLAB (The MathWorks Inc., Natick, MA, USA) using the EasySpin program package for EPR [29].

X-ray powder diffractions (XRPD) were recorded in the 5 ÷ 32° 2θ range using Shimadzu XRD-7000 powder diffractometer (CuKα tube with Ni filter, Bragg-Brentano scheme with a vertical θ-θ goniometer, OneSight SSD-detector).

The structures of the complexes were optimized by a spin-unrestricted DFT method (spin polarization—5) using the Amsterdam density functional [30,31] program with a gradient exchange functional GGA (BP86—Becke [32] and Perdew [33,34]). Triple zeta polarized (TZP) basis sets and the no frozen core approximation were used in all calculations. The initial positions of atoms were taken from the X-ray structure analysis. The frequency analysis was carried out to check the nature of the stationary points (BP86, TZP) and imaginary frequencies were not found. The calculation of the g-tensor [35] was made using Spin-Orbit ZORA [36,37,38] approximation with a hybrid functional PBE0 [39,40]. The zero-field parameters were obtained using the method proposed by van Wüllen and coworkers [41,42]. The electronic excitations were calculated using the TD-DFT method [43,44] (BP86, B3LYP [45], and CAM-B3LYP [46] functionals, TZP) and additionally with the spin-flip approximation [47,48]. The quantum theory of atoms in molecules (QTAIM) [49] extended transition state method with the natural orbitals for chemical valence theory (ETS-NOCV), and the non-covalent interaction (NCI) calculations [50,51,52,53,54,55,56] were carried out to investigate the non-covalent interactions using the ADF program (BP86, TZP). The [H_2_DPEphos]^2+^ cation and [MnX_4_]^2−^ anion were used as fragments to calculate ETS-NOCV.

FT-IR spectra were recorded on a Bruker Vertex 80 spectrometer at ambient temperature.

Differential scanning calorimetric measurements were performed using a heat flow measurement method using a Netzsch TG 209 F1 calorimeter with a heating rate of 10 °C min^−1^, in a He flow of 30 mL min^−1^.

### 3.1. Structural Descriptions

The single-crystal X-ray diffraction analysis reveals that complexes **1** and **2** crystallize in centrosymmetric space group *P*-1. The unit cell contains two [H_2_DPEphos]^2+^ cations and two [MnX_4_]^2−^ anions that are not related by symmetry and have slightly different geometry parameters (*Z*′ = 2). The inversion center located not on the fragments completes the unit cell (*Z* = 4). Unfortunately, the presence of a center of symmetry makes the presence of triboluminescence unlikely. Complexes consist of isolated [MnX_4_]^2−^ anions surrounded by the [H_2_DPEphos]^2+^ cations. Every Mn(II) atom in **1** and **2** is four coordinated by the four halide atoms. The positions of the manganese and halide ions are disordered in the crystal. The disorder of [MnX_4_]^2^^−^ fragments can be resulted from the thermal (vibration) motion of atoms and/or the deviation of the nuclei position in each unit cell from the position averaged for the whole crystal. The coordination geometry around the Mn(II) can be described as a distorted tetrahedron with the Mn-X distances (and X-Mn-X angles) varying in the range of 2.320–2.680 Å (105.0–115.9°) for **1** and 2.295–2.406 Å (102.4–116.6°) for **2**. The dihedral angles between phenyl rings of the diphenyl ether fragment are 118.0° and 118.8° for **1** and 117.8° and 118.2° for **2** which is close to values for diphenyl ether (Ph_2_O, CCDC RAFFIO) (118.3°) and DPEphos (CCDC GAJRIT) (118.9°). The C-O-C angles are 69.5° and 70.1° for **1** and 68.1° and 68.8° for **2** whereas the C-O-C angle is 75.6° for Ph_2_O and 67.2° for DPEphos. Along with increasing the C-O-C angles in **1** and **2** relative to the corresponding DPEphos angle, the P-P distance also significantly increases from 4.876 Å for DPEphos to 5.193 Å and 5.218 Å for **2** and 5.247 Å and 5.269 Å for **1**. The P-Mn-P angles are 70.2° and 71.0° for **1** and 70.9° and 71.2° for **2**.

The non-covalent CH…X(Mn) and PH…X(Mn) interactions between [H_2_DPEphos]^2+^ and [MnX_4_]^2−^ fragments form a 3D intermolecular contacts framework. The shortest PH…X(Mn) distance is 2.532 Å for **1** and 2.465 Å for **2** which is close to the distances in the PH_4_X compounds [57,58]. The presence of PH…X(Mn) and CH…X(Mn) interactions may be one of the reasons resulting in the disordering of the [MnX_4_]^2−^ anions following the PH…X(Mn) and CH…X(Mn) vibrations (see below).

According to the obtained luminescence and magnetic properties for **1** and **2**, the different properties for the non-equivalent cations and anions, and the disordering atoms were not experimentally noticed. The non-equivalence and disordering probably lead to the broadening of the luminescence and EPR spectral lines.

### 3.2. Photophysical Properties

**Figure 1:**
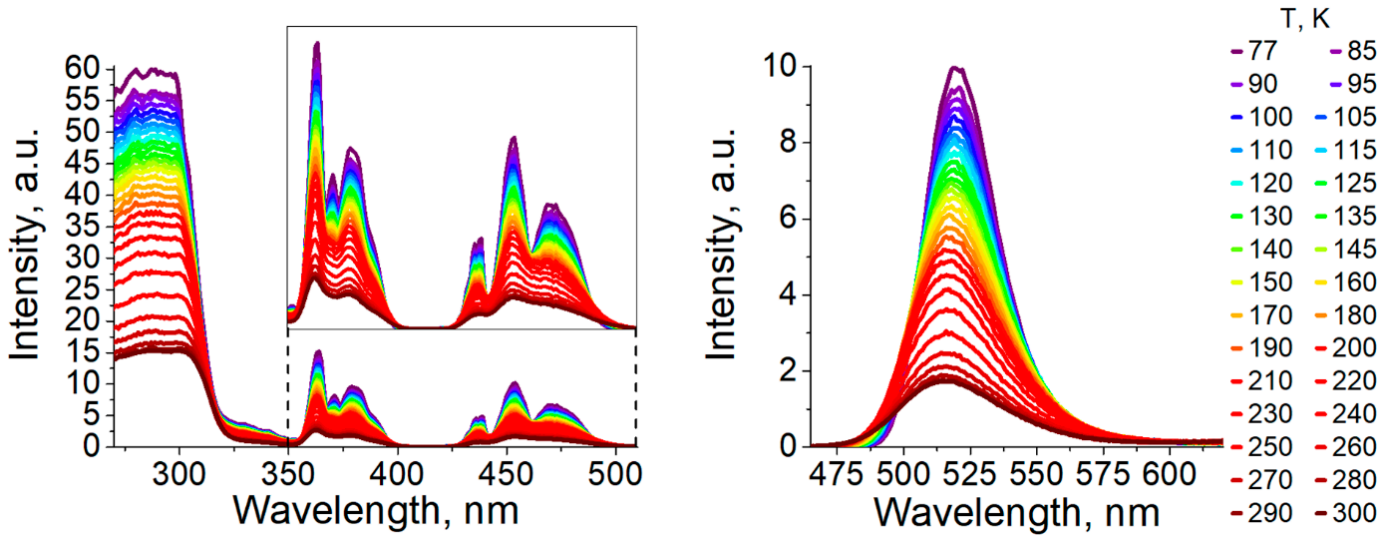
Temperature dependences of the photoluminescence excitation spectra (λ_Em_ = 520 nm) and photoluminescence spectra (λ_Ex_ = 453 nm) of **1**.

**Figure 2:**
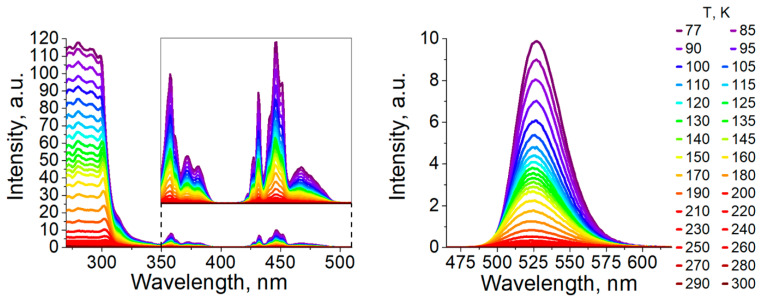
Temperature dependences of the photoluminescence excitation spectra (λ_Em_ = 527 nm) and photoluminescence spectra (λ_Ex_ = 447 nm) of **2**.

The solid samples of **1** and **2** exhibit broadband temperature-dependent photoluminescence in the visible region (Figure 1 and Figure 2). The photophysical data for **1** and **2** are collected in Table 2. The luminescence maximum λ_max_ = 515 nm and 523 nm at 300 K for **1** and **2**, respectively, red-shifts to λ_max_ = 520 nm and 527 nm at 77 K for **1** and **2**, respectively. At the same time, the narrowing of the spectral line and increasing of the photoluminescence integral intensity is observed. Such temperature behavior is typical for the tetrahalomanganates(II) [14]. It should be noted that the luminescence maximum for both complexes is the wavelength-excitation independent in the energy range λ_Ex_ = 240–500 nm. However, the quantum yield of the luminescence Φ_PL_ decreases with the energy excitation decreasing from 2% under excitation λ_Ex_ = 300 nm to ≈0.1% under λ_Ex_ = 447 nm for **2** and from 60% under λ_Ex_ = 300 nm to 42% under λ_Ex_ = 453 nm for **1** at 300 K. Such behavior can be associated with the charge transfer process realized between the [H_2_DPEphos]^2+^ cation and the [MX_4_]^2−^ anion through PH…X(Mn) and CH…X(Mn) hydrogen bonds (see below).

The photoluminescence excitation spectra of both complexes (Figure 1 and Figure 2) consist of the well-resolved lines in the range 350–500 nm and partially resolved broadband at higher energy. The analysis of the excitation spectra is carried out using the Tanabe–Sugano approach [59] with the tetrahedral/octahedral symmetry approximation to estimate the crystal field splitting parameter (*Dq_tet_*) and Racah parameter (*B*) [60,61]. The reduction of the parameter *B*^Mn^ from the free ion value *B*_0_^Mn^ = 923 cm^−1^ [62] is observed (β = *B*/*B*_0_^Mn^ = 0.69 and 0.66 for **1** and **2**, respectively). Such reduction indicates the formation of the covalent bonds involving manganese orbitals and/or that the effective positive charge on the metal decreased. Contrariwise to the *B* parameter, the *Dq_tet_* parameter for **1** is less than for **2**, which is in good agreement with the ligand field theory. Based on the obtained data in combination with the EPR data (see below), the observed luminescence originated from the ^4^*T*_1_(**G**)→^6^*A*_1_(**S**) transition and can be ascribed to the phosphorescence. The efficiency of the [MnX_4_]^2−^ phosphorescence depends on the intersystem crossing processes caused by the presence of the halide ions. The spin-orbit coupling constant for Br is significantly bigger than for Cl, leading to the intersystem crossing process for **1** being bigger than for **2**.

**Figure 3:**
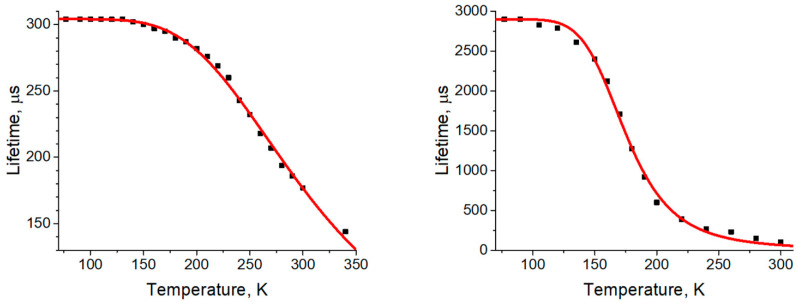
Temperature dependences of the photoluminescence lifetime of **1** (λ_Ex_ = 453 nm, λ_Em_ = 520 nm, left) and **2** (λ_Ex_ = 447 nm, λ_Em_ = 527 nm, right).

The temperature dependences of the luminescence decays are obtained to estimate the activation energy for the thermal quenching process. The luminescence decays are described by the monoexponential function for complexes **1** and **2** in 77–300 K temperature range:

(1) $$I\left(t\right)=I_{0}*\exp\left(-\frac{t}{\tau }\right)$$

where $I_{0}$ is the intensity at t = 0 and  τ is the lifetime. It should be noted that the luminescence lifetime is wavelength-excitation independent. The luminescence lifetimes increase from 177 μs and 100 μs at 300 K to 304 μs and 2900 μs at 77 K for **1** and **2**, respectively. The temperature dependences of  τ are fitted by the following equation (Figure 3):

(2) $$\tau \left(T\right)=\tau_{0}/\left(1+C*\exp\left(-\frac{\Delta E}{T}\right)\right)$$

where $\tau_{0}$ is the temperature-independent radiative lifetime, C is the dimensionless parameter which can be seen as the ratio between the non-radiative and radiative probabilities, and ΔE is the activation energy for the thermal quenching process which is equal to the ΔE = 1300 K (900 cm^−1^) and 1600 K (1100 cm^−1^) for **1** and **2**, respectively. The thermal behavior depends on the potential energy parameters. The thermal quenching process is originated from the crossing of the excited state potentials with ground state potential following energy dissipation via the vibrations. At least three different pathways of the non-radiative transitions for the [MnX_4_]^2−^ core can be highlighted. The non-radiative relaxation can occur between the ^4^*T*_1_(**G**) and ^6^*A*_1_(**S**) states directly and through ^4^*T*_2_(**G**) and charge transfer states. Together with the above-mentioned increasing of the intersystem crossing process probability, the ^4^*T*_2_(**G**) level is closer to ^4^*T*_1_(**G**) in **1** than in **2** (Table S11). These can explain the observed different thermal behavior of the investigated complexes. In addition, the PH…X(Mn) vibrations can play an important role in the quenching processes. The two main PH…X(Mn) vibrations have energy ΔE = 2133 cm^−1^ (777 km/mol) and 2333 cm^−1^ (349 km/mol) for **2** and ΔE = 2155 cm^−1^ (679 km/mol) and 2370 cm^−1^ (240 km/mol) for **1**. It can be assumed that the above-mentioned vibrations result in the high ratio of the non-radiative transition rates to radiative transition rate and, consequently, the unexpected short luminescence lifetime and low quantum yield for **2** at 300 K.

For deeper investigation of the PH…X(Mn) interactions, the quantum theory of atoms in molecules (QTAIM) and the extended transition state method with natural orbitals for chemical valence theory (ETS-NOCV) calculations were carried out for **1** and **2**. According to the QTAIM calculation (Figure S8), the (3, −1) bond critical points exist between hydrogen and halide ions, including the bonds between PH and X(Mn) fragments. This approach gives a quantum mechanical description of the topology of the weak bonds in molecules. The detailed information about weak interactions in **1** and **2** was obtained using the ETS-NOCV approach with non-covalent interaction (NCI) analysis. The calculation of the deformation density Δ*ρ_i_* shows that the interaction between the [H_2_DPEphos]^2+^ cation and the [MnX_4_]^2−^ anion results in the decreasing of the density *ρ_i_* on the X and H nuclei of the PH…X(Mn) fragments and the increasing of the density *ρ_i_* in the area between the (P)H and X(Mn) ions. The NCI analysis shows the presence of the PH…X(Mn) attractive interactions both in **1** and **2** (Figure S9). Obtained results indicate the possibility of the occurrence of the charge transfer processes between [H_2_DPEphos]^2+^ cation and the [MX_4_]^2−^ anion.

**Figure 4:**
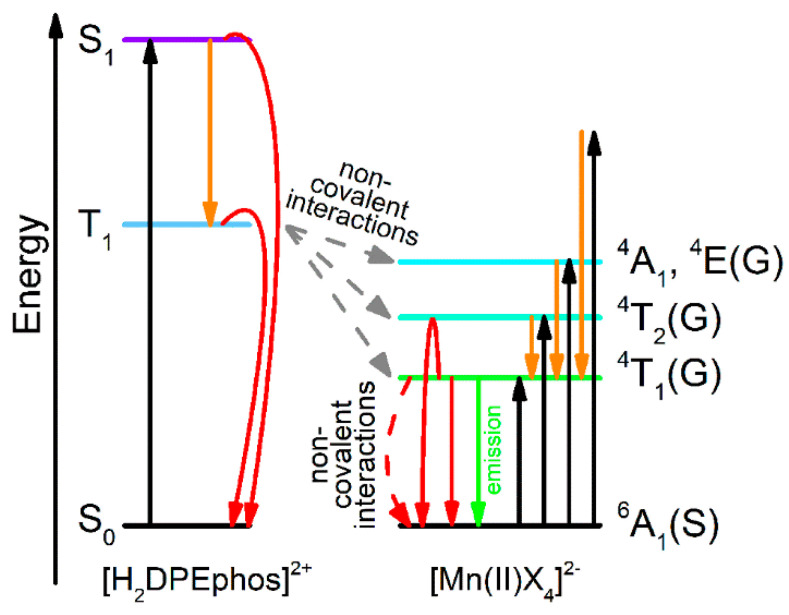
Representation of the energy absorption, transfer, and emission processes in **1** and **2** (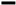
—absorption, 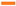
—internal conversion and intersystem crossing transition, 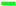
—emission, 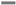
—transfer from [H_2_DPEphos]^2+^ to [MnX_4_]^2−^, 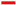
—non-radiative relaxation).

It is interesting to compare the properties of the obtained complexes and the properties of the tetrahedral manganese(II) with bis[2-(diphenylphosphino)phenyl]ether oxide (DPEPO) ligand [15] crystallizing in the same *P*-1 space group. The [MnBr_2_(DPEPO)] and [MnCl_2_(DPEPO)] complexes exhibit the intense room-temperature luminescence with the maximum at λ = 502 nm and 507 nm, quantum yield Φ_PL_ = 70% and 32%, and luminescence lifetime τ = 0.5 ms and 2.2 ms, respectively. Authors assume that the high efficiency of luminescence is caused by the effective intersystem crossing process and the energy effectively transfers from DPEPO ligand to Mn(II) ion. The close situation can be realized in complexes **1** and **2**. Indeed, the quantum-chemical calculations obtained for [H_2_DPEphos]^2+^ cation, [H_2_DPEphos][ZnCl_4_], and [H_2_DPEphos][ZnBr_4_] show that the phosphorus and hydrogen (PH…X(Mn)) orbitals involved in the electronic excitations with the high oscillation strength in the energy range up to 5 eV (248 nm). The energy of the singlet-singlet transition (CAM-B3LYP functional) with high oscillation strength equals to ΔE(π1π) = 4.46 eV (278 nm) and ΔE(π1π) = 4.59 eV (270 nm) for [H_2_DPEphos][ZnCl_4_] and [H_2_DPEphos][ZnBr_4_] (Table S13), respectively. Whereas the energy of the singlet-triplet transition equals to ΔE(π3π) = 3.31 eV (375 nm) and ΔE(π3π) = 3.23 eV (384 nm) for [H_2_DPEphos][ZnCl_4_] and [H_2_DPEphos][ZnBr_4_] (Table S13), respectively. The singlet-singlet and singlet-triplet transitions are calculated for the cations with the atom position taken from the [H_2_DPEphos][ZnCl_4_] and [H_2_DPEphos][ZnBr_4_] to investigate the changes of the transition energies in the [H_2_DPEphos]^2+^ cation resulting from the interaction with the [ZnX_4_]^2−^ anion (Table S14). For both cation geometries, the transition energies are close and equal to ΔE(π1π) = 4.66 eV (266 nm) and ΔE(π3π) = 3.23 eV (384 nm). The interaction of the [H_2_DPEphos]^2+^ cation with the [ZnX_4_]^2−^ anion results in the decreasing of the ΔE(π1π) and ΔE(π1π−π3π) energies. Nevertheless, the ΔE(π1π−π3π) energy gap is still higher than 5000 cm^−1^ and equals to 1.15 eV (9325 cm^−1^) and 1.36 eV (10,987 cm^−1^) for [H_2_DPEphos][ZnCl_4_] and [H_2_DPEphos][ZnBr_4_] (Table S13), respectively. According to the Reinhoudt’s empirical rule [15], the intersystem crossing process is effective in the [H_2_DPEphos]^2+^ cation with ΔE(π1π−π3π) > 5000 cm^−1^. At the same time, the energies of the **G** state sublevels of the manganese(II) ion for **1** and **2** are less than 2.92 eV (425 nm), lower than the estimated ΔE(π3π) energy. Therefore, the energy can be transferred from the [H_2_DPEphos]^2+^ cation to the Mn(II) ion of [MX_4_]^2−^ through PH…X(Mn) hydrogen bonds [63]. The above-mentioned mechanism can explain the wavelength-excitation dependence of the luminescence quantum yield while the luminescence maximum, linewidth, and lifetime do not exhibit wavelength-excitation dependence and the reasons for the luminescence thermal quenching features of both complexes. The possible energy absorption and emission processes and energy migration pathways are shown in Figure 4.

### 3.3. Electron Paramagnetic Resonance

**Figure 5:**
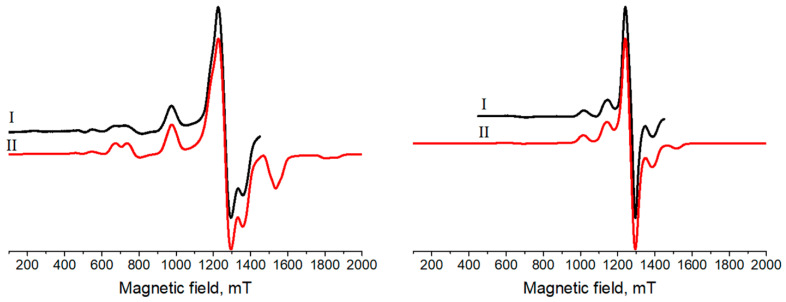
Q-band EPR spectra of solid **1** (**left**) and **2** (**right**) at 300 K. (I—experimental, II—the best fit of the experimental data).

The Q-band EPR spectra of polycrystalline complexes **1** and **2** were recorded at room temperature under non-saturated conditions (Figure 5). The EPR spectra were described by the spin-Hamiltonian:

(3) $$\hat{H}=\beta *B*g*\hat{S}+D\left[\hat{S_{z}^{2}}-\frac{S*\left(S+1\right)}{3}\right]+E\left[\hat{S_{x}^{2}}-\hat{S_{y}^{2}}\right]$$

with parameters presented in Table 3., data were analyzed using the EasySpin program [29]. The hyperfine interaction for Mn(II) ion is ignored due to the absence of the resolved line that is typical for this ion in the spectra. According to the obtained data, the ground state of the Mn(II) is an orbital singlet ^6^**S**_5/2_ (S = 5/2, L = 0) and the crystal field splitting does not remove the degeneracy of orbital levels. The external magnetic field can remove the degeneracy of spin levels. However, the interaction of the unpaired electrons can result in the lifting of spin levels degeneracy without an external magnetic field. This is so-called zero-field splitting (ZFS) which is usually defined via D and E parameters. The two different types of interaction between unpaired electrons contribute to the total zero-field splitting $D_{tot}=D_{SS}+D_{SOC}$. The first interaction is a spin–spin dipole–dipole $D_{SS}$ interaction between metal ions. The second is a spin-orbit coupling $D_{SOC}$ term. The last part consists of four different types of excitations presented in the one-electron approximation as α→β (spin-flip excitation corresponding to the spin-pairing ∆S = –1) and the charge-transfer transitions β→α, β→β, and α→α [64]. The first term (α→β) mainly defines the efficiency of the luminescence and corresponding forbidden *d*–*d* transitions. Other terms can play an important role in the quenching processes by energy dissipation. The complex **1** is characterized by the relatively high value of the parameter D with the contribution of the α→β term compared with other charge-transfer terms and $D_{SS}$ term. Whereas the complex **2** is characterized by the significantly less value of the parameter D and the contribution of the charge-transfer terms being predominant. This fact can explain the observed difference of the luminescence efficiency between **1** and **2** additionally to the reasons mentioned in the photophysical properties section.

## 4. Conclusions

The luminescent organic–inorganic halomanganate(II) compounds with P,P’-diprotonated bis(2-diphenylphosphinophenyl)ether have been synthesized and investigated. The ground state of the Mn(II) ions in the complexes is an orbital singlet ^6^**S**_5/2_ (^6^*A*_1_). The luminescence of complexes is caused by the ^4^*T*_1_(**G**)→^6^*A*_1_(**S**) transition under 240–500 nm excitation energies. The low excitation energy (~500–300 nm) leads to the (M + X) transition due to the spin-orbit coupling and the spin-flip processes in the [MnX_4_]^2−^ anion. The EPR spectrum of **1** is characterized by the zero-field splitting with the significant contribution of the spin-orbit spin-flip α→β term. The spin-flip α→β term of **2** is significantly less and this can explain the lower excitation of the luminescence for **2** in the low energy excitation range.

Under higher energies (>300 nm), the [H_2_DPEphos]^2+^ cation can absorb the light due to the π1π transitions involving the orbitals of the PH groups connected to the manganese(II) through the PH…X(Mn) bonds. Then the intersystem crossing occurs from the π1π state to the π3π state also containing the PH group orbitals followed by the charge transfer process from [H_2_DPEphos]^2+^ cation to [MnX_4_]^2−^ anion through the PH…X(Mn) bonds. In this case, the two different excitation pathways ((M + X) and π1π→π3π→(M+X) transitions) can take place. The PH…X(Mn) bond plays an important role not only in the charge transfer processes but also in the luminescence quenching processes for both above-mentioned Mn(II) excitation pathways.

## Figures and Tables

**Table 1:** Photophysical data (peak position and luminescence quantum yield) for [MnX_4_]^2−^-containing compounds.

Cation	Halide	Quantum Yield, %	λ_Em_, nm	Ref.
Ph_3_P-Ph	Br	98	516	[16]
Ph_3_P-Ph	Cl	40	522	[17]
Ph_3_P-Benzyl	Br	90	510	[13]
Ph_3_P-Benzyl	Cl	32	509	[13]
Ph_3_P-Et	Br	91	522	[13]
Ph_3_P-Et	Cl	29	524	[13]
Ethylenebis- Ph_3_P	Br	95	517	[18]
Ph(Me)_3_N	Br	76	520	[2]
Ph(Me)_3_N	Cl	89	522	[2]
Et_4_N	Br	86	516	[2]
Et_4_N	Cl	75	518	[2]

**Table 2:** Photophysical data for polycrystalline complexes **1** and **2**.

	1 (Br)	2 (Cl)
λ_max_(300 K) [nm] ^a^	515	523
*τ*(300 K) [µs] ^a^	177	100
Φ_PL_(300 K) [%] ^a^	42	≈0.1
*k*_r_(300 K) [s^–1^] ^a,b^	2.4 × 10^3^	≈1.0 × 10^1^
*k*_nr_(300 K) [s^–1^] ^a,c^	3.3 × 10^3^	≈1.0 × 10^4^
Φ_PL_(300 K) [%] ^e^	60	2
*k*_r_(300 K) [s^–1^] ^e,b^	3.4 × 10^3^	2.0 × 10^2^
*k*_nr_(300 K) [s^–1^] ^e,c^	2.3 × 10^3^	9.8 × 10^3^
Chromaticity (300 K) [x, y] ^d^	0.164; 0.750	0.259; 0.569
λ_max_(77 K) [nm] ^a^	520	527
*τ*(77 K) [µs] ^a^	304	2900
Φ_PL_(77 K) [%] ^a^	≈100	40
*k*_r_(77 K) [s^–1^] ^a,b^	3.3 × 10^3^	1.4 × 10^2^
*k*_nr_(77 K) [s^−1^] ^a,c^	<3.3 × 10^2^	2.1 × 10^2^
Φ_PL_(77 K) [%] ^e^	≈100	≈100
*k*_r_(77 K) [s^–1^] ^e,b^	3.3 × 10^3^	3.4 × 10^2^
*k*_nr_(77 K) [s^−1^] ^e,c^	<3.3 × 10^2^	<3.4 × 10^1^
Chromaticity (77 K) [x, y] ^d^	0.216; 0.656	0.205; 0.744
ΔE [cm^−1^]	900	1100
*10Dq_tet_* [cm^−1^]	1730	1960
*B* [cm^−1^]	638	608

^a^ Excitation wavelengths are λ_Ex_ = 453 nm and 447 nm for **1** and **2,** respectively; ^b^ Radiative decay rate: *k*_r_ = Φ_PL_/*τ*; ^c^ Non-radiative decay rate: *k*_nr_ = *τ*^−1^ − *k*_r_; ^d^ CIE 1931 color space; ^e^ Excitation wavelength is λ_Ex_ = 300 nm.

**Table 3:** Experimental and calculated EPR parameters for polycrystalline complexes **1** and **2**.

	1	2
S	5/2	5/2
$g_{exp}\;$ ^a^	2.005	2.000
$g_{calc\;}$ ^a^	2.008	2.003
${\left|D\right|}_{exp}$[MHz] ^b^	4200	1760
${\left|D\right|}_{calc}$[MHz] ^b^	4800	2007
${\left|E\right|}_{exp}$[MHz] ^b^	903	380
${\left|E\right|}_{calc}$[MHz] ^b^	525	356

^a^g—g-tensor, ^b^D and E—zero-field splitting (ZFS) parameters.

## Supplementary Materials

The following are available online at https://www.mdpi.com/article/10.3390/ijms22136873/s1. Crystallographic data, X-ray powder diffraction data, IR spectra, calorimetric data, and DFT calculation data. CCDC: 2078076 and 2078077.

## References

[1] Hausmann D., Kuzmanoski A., Feldmann C. (2016). MnBr2/18-crown-6 coordination complexes showing high room temperature luminescence and quantum yield. Dalton Trans..

[2] Morad V., Cherninkh I., Pottschacher L., Shynkarenko Y., Yakunin S., Kovalenko M.V. (2019). Manganese(II) in tetrahedral halide environment: Factors governing bright green luminescence. Chem. Mater..

[3] Etaiw S., Marie H. (2019). Sonochemical nanostructure of Mn(II) supramolecular complex: X-ray structure, sensing and photocatalytic properties. Sens. Actuators B Chem..

[4] Godfrey S.M., McAuliffe C.A., Ndifon P.T., Pritchard R.G. (1993). Controlled reaction of molecular oxygen with [MnI_2_(PPh_2_Me)_2_] to form the mixed phosphine–phosphine oxide complex [MnI_2_(OPPh_2_Me)(PPh_2_Me)] and the Bis(phosphine oxide) complex [MnI_2_(OPPh_2_Me)_2_]. J. Chem. Soc. Dalton Trans..

[5] McAuliffe C.A., Godfrey S.M., Mackie A.G., Pritchard R.G. (1992). The reaction of coarse-grain manganese powder with diiodotrimethylphosphorane to form [Mn(PMe_3_)I_2_]_n_, which reacts with trace quantities of molecular oxygen to form the mixed (+2/+3) oxidation state complex [Mn_2_(PMe_3_)_3_I_5_]PMe_3_. J. Chem. Soc. Chem. Commun..

[6] Davydova M.P., Bauer I.A., Brel V.K., Rakhmanova M.I., Yu I. (2020). Bagryanskaya and A. V. Artem’ev, Manganese(II) thiocyanate complexes with bis(phosphine oxide) ligands: Synthesis and excitation wavelength-dependent multicolor luminescence. Eur. J. Inorg. Chem..

[7] Berezin A.S., Vinogradova K.A., Nadolinny V.A., Sukhikh T.S., Krivopalov V.P., Nikolaenkova E.B., Bushuev M.B. (2018). Temperature- and excitation wavelength-dependent emission in a manganese(II) complex. Dalton Trans..

[8] Stalzer M.M., Lohr T.L., Marks T.J. (2018). Synthesis, characterization, and thermal properties of N-alkyl beta-diketiminate manganese complexes. Inorg. Chem..

[9] Wang R.H., Gao E.Q., Hong M.C., Gao S., Luo J.H., Lin Z.Z., Han L., Cao R. (2003). A three-dimensional manganese(II) complex exhibiting ferrimagnetic and metamagnetic behaviors. Inorg. Chem..

[10] Bouchoucha A., Terbouche A., Bourouina A., Djebbar S. (2014). New complexes of manganese(II), nickel(II) and copper(II) with derived benzoxazole ligands: Synthesis, characterization, DFT, antimicrobial activity, acute and subacute toxicity. Inorg. Chim. Acta.

[11] Mabad B., Cassoux P., Tuchagues J.P., Hendrickson D.N. (1986). Manganese(II) complexes of polydentate schiff-bases. 1. Synthesis, characterization, magnetic-properties, and molecular-structure. Inorg. Chem..

[12] Eichhofer A., Lebedkin S. (2018). 1D and 3D polymeric manganese(II) thiolato complexes: Synthesis, structure, and properties of ^3^[Mn_4_(SPh)_8_] and ^1^[Mn(SMes)_2_]. Inorg. Chem..

[13] Qin Y.Y., She P.F., Huang X.M., Huang W., Zhao Q. (2020). Luminescent manganese(II) complexes: Synthesis, properties and optoelectronic applications. Coord. Chem. Rev..

[14] Berezin A.S., Davydova M.P., Samsonenko D.G., Sukhikh T.S., Artem’ev A.V. (2021). A family of brightly emissive homo- and mixed-halomanganates(II): The effect of halide on optical and magnetic properties. J. Lumin..

[15] Chen J., Zhang Q., Zheng F.K., Liu Z.F., Wang S.H., Wu A.Q., Guo G.C. (2015). Intense photo- and tribo-luminescence of three tetrahedral manganese(II) dihalides with chelating bidentate phosphine oxide ligand. Dalton Trans..

[16] Xu L.-J., Sun C.-Z., Xiao H., Wu Y., Chen Z.-N. (2017). Green-light-emitting diodes based on tetrabromide manganese(II) complex through solution process. Adv. Mater..

[17] Ben-Akacha A., Zhou C., Chaaban M., Beery D., Lee S., Worku M., Lin X., Westphal R., Ma B. (2021). Mechanochemical synthesis of zero dimensional organic-inorganic metal halide hybrids. ChemPhotoChem.

[18] Xu L.-J., Lin X., He Q., Worku M., Ma B. (2020). Highly efficient eco-friendly X-ray scintillators based on an organic manganese halide. Nat. Commun..

[19] Sun M.-E., Li Y., Dong X.-Y., Zang S.-Q. (2019). Thermoinduced structural-transformation and thermochromic luminescence in organic manganese chloride crystals. Chem. Sci..

[20] Gong L.-K., Hu Q.-Q., Huang F.-Q., Zhang Z.-Z., Shen N.-N., Hu B., Song Y., Wang Z.-P., Du K.-Z., Huang X.-Y. (2019). Efficient modulation of photoluminescence by hydrogen bonding interactions between inorganic [MnBr_4_]^2−^ anions and organic cations. Chem. Commun..

[21] Ratajczak H. (1972). Charge-transfer properties of the hydrogen bond. I. Theory of the enhancement of dipole moment of hydrogen-bonded systems. J. Phys. Chem..

[22] Christodouleas N., McGlynn S.P. (1964). Energy transfer in charge-transfer complexes. III. Intersystem crossing. J. Chem. Phys..

[23] Trotter P.J. (1968). Internal compression effects. II. Frequency and intensity changes in charge-transfer and hydrogen-bonded complex spectra. J. Chem. Phys..

[24] Rigaku (2020). CrysAlisPro Software System, Version V1.171.41.83a, Rigaku Oxford Diffraction. http://www.rigaku.com.

[25] Sheldrick G.M. (2015). SHELXT-Integrated space-group and crystal-structure determination. Acta Crystallogr. A Found. Adv..

[26] Dolomanov O.V., Bourhis L.J., Gildea R.J., Howard J.A., Puschmann H. (2009). OLEX2: A complete structure solution, refinement and analysis program. J. Appl. Crystallogr..

[27] Sheldrick G.M. (2015). Crystal structure refinement with SHELXL. Acta Crystallogr. Sect. C Struct. Chem..

[28] Artem’ev A.V., Davydova M.P., Berezin A.S., Ryzhikov M.R., Samsonenko D.G. (2020). Dicopper(I) paddle-wheel complexes with thermally activated delayed fluorescence adjusted by ancillary ligands. Inorg. Chem..

[29] Stoll S., Schweiger A. (2006). EasySpin, a comprehensive software package for spectral simulation and analysis in EPR. J. Magn. Reson..

[30] Te Velde G.T., Bickelhaupt F.M., Baerends E.J., Guerra C.F., Van Gisbergen S.J.A., Snijders J.G., Ziegler T. (2001). Chemistry with ADF. J. Comput. Chem..

[31] (2019). ADF2019, ADF2019.3, SCM, Theoretical Chemistry, Vrije Universiteit, Amsterdam, The Netherlands. http://www.scm.com.

[32] Becke A.D. (1988). Density-functional exchange-energy approximation with correct asymptotic-behavior. Phys. Rev. A.

[33] Perdew J.P. (1986). Density-functional approximation for the correlation-energy of the inhomogeneous electron-gas. Phys. Rev. B.

[34] Perdew J.P. (1986). Correction. Phys. Rev. B.

[35] van Lenthe E., van der Avoird A., Wormer P.E.S. (1997). Density functional calculations of molecular g-tensors in the zero-order regular approximation for relativistic effects. J. Chem. Phys..

[36] van Lenthe E., Baerends E.J., Snijders J.G. (1993). Relativistic regular 2-component Hamiltonians. J. Chem. Phys..

[37] van Lenthe E., Baerends E.J., Snijders J.G. (1994). Relativistic total-energy using regular approximations. J. Chem. Phys..

[38] van Lenthe E., Ehlers A.E., Baerends E.J. (1999). Geometry optimizations in the zero order regular approximation for relativistic effects. J. Chem. Phys..

[39] Grimme S. (2004). Accurate description of van der Waals complexes by density functional theory including empirical corrections. J. Comput. Chem..

[40] Ernzerhof M., Scuseria G. (1999). Assessment of the Perdew-Burke-Ernzerhof exchange-correlation functional. J. Chem. Phys..

[41] van Wüllen C. (2009). Magnetic anisotropy from density functional calculations. Comparison of different approaches: Mn_12_O_12_ acetate as a test case. J. Chem. Phys..

[42] Schmitt S., Jost P., van Wüllen C. (2011). Zero-field splittings from density functional calculations: Analysis and improvement of known methods. J. Chem. Phys..

[43] van Gisbergen S.J.A., Snijders J.G., Baerends E.J. (1999). Implementation of time-dependent density functional response equations. Comput. Phys. Commun..

[44] Wang F., Ziegler T. (2005). A simplified relativistic time-dependent density-functional theory formalism for the calculations of excitation energies including spin-orbit coupling effect. J. Chem. Phys..

[45] Stephens P.J., Devlin F.J., Chabalowski C.F., Frisch M.J. (1994). Ab initio calculation of vibrational absorption and circular dichroism spectra using density functional force fields. J. Phys. Chem..

[46] Yanai T., Tew D.P., Handy N.C. (2004). A new hybrid exchange–correlation functional using the Coulomb-attenuating method (CAM-B3LYP). Chem. Phys. Lett..

[47] Wang F., Ziegler T. (2004). Time-dependent density functional theory based on a noncollinear formulation of the exchange-correlation potential. J. Chem. Phys..

[48] Wang F., Ziegler T. (2005). The performance of time-dependent density functional theory based on a noncollinear exchange-correlation potential in the calculations of excitation energies. J. Chem. Phys..

[49] Bader R.F.W. (1991). A quantum theory of molecular structure and its applications. Chem. Rev..

[50] Mitoraj M., Michalak A., Ziegler T. (2009). A combined charge and energy decomposition scheme for bond analysis. J. Chem. Theory Comput..

[51] Rodríguez J.I. (2013). An efficient method for computing the QTAIM topology of a scalar field: The electron density case. J. Comput. Chem..

[52] Rodríguez J.I., Bader R.F.W., Ayers P.W., Michel C., Götz A.W., Bo C. (2009). A high performance grid-based algorithm for computing QTAIM properties. Chem. Phys. Lett..

[53] Johnson E.R., Keinan S., Mori-Sánchez P., Contreras-García J., Cohen A.J., Yang W. (2010). Revealing noncovalent interactions. J. Am. Chem. Soc..

[54] Contreras-García J., Johnson E.R., Keinan S., Chaudret R., Piquemal J.-P., Beratan D.N., Yang W. (2011). NCIPLOT: A Program for plotting noncovalent interaction regions. J. Chem. Theory Comput..

[55] Aarabi M., Gholami S., Grabowski S.J. (2020). S−H…O and O−H…O hydrogen bonds-comparison of dimers of thiocarboxylic and carboxylic acids. ChemPhysChem.

[56] Poater J., Gimferrer M., Poater A. (2018). Covalent and ionic capacity of mofs to sorb small gas molecules. Inorg. Chem..

[57] Fluck E., Svara J., Neumuller B., Riffel H., Thurn H. (1986). Darstellung und struktur von methylphosphoniumchlorid. Z. Anorg. Allg. Chem..

[58] Schroeder L.W., Rush J.J. (1971). Neutron diffraction study of the structure and thermal motion of phosphonium bromide. J. Chem. Phys..

[59] Tanabe Y., Sugano S. (1954). On the absorption spectra of complex ions. I. J. Phys. Soc. Jpn..

[60] Racah G. (1943). Theory of Complex Spectra. III. Phys. Rev..

[61] Racah G. (1949). Theory of Complex Spectra. IV. Phys. Rev..

[62] Liu X., Dronskowski R., Glaum R., Tchougréeff A.L. (2010). Experimental and quantum-chemical investigations of the UV/Vis absorption spectrum of manganese carbodiimide, MnNCN. Z. Anorg. Allg. Chem..

[63] Gautier R., Paris M., Massuyeau F. (2020). Hydrogen bonding and broad-band emission in hybrid zinc halide phosphors. Inorg. Chem..

[64] Duboc C., Phoeung T., Zein S., Pécaut J., Collomb M.N., Neese F. (2007). Origin of the zero-field splitting in mononuclear octahedral dihalide Mn-II complexes: An investigation by multifrequency high-field electron paramagnetic resonance and density functional theory. Inorg. Chem..

