# Supplementary material for: A Halomanganates(II) with P,P’-Diprotonated Bis(2-Diphenylphosphinophenyl)ether: Wavelength-Excitation Dependence of the Quantum Yield and Role of the Non-Covalent Interactions
Source: Int J Mol Sci. 2021 Jun 26;22(13):6873. doi: 10.3390/ijms22136873 (PMC8268785; doi:10.3390/ijms22136873)
Supplement: Supplementary file 1 [file ijms-22-06873-s001.zip › ijms-1208742-supplementary.pdf]

Electronic Supplementary Information

A halomanganates(II) with P,P'-diprotonated bis(2-diphenylphosphinophenyl)ether: wavelength-excitation dependence of the quantum yield and role of the hydrogen bonds.

Alexey S. Berezin

Table S1. X-Ray crystallographic data for **1** and **2**.

**Compound**	**1**	**2**
Formula	$C_{36}H_{30}Br_4MnOP_2$	$C_{36}H_{30}Cl_4MnOP_2$
$D_{calc.}$/ g cm^{-3}	1.679	1.408
μ/mm^{-1}	9.184	6.999
Formula Weight	915.12	737.28
Colour	fluorescent colourless	clear colourless
Shape	prism	prism
Size/mm^3	0.20×0.12×0.07	0.13×0.09×0.08
T/K	123.01(10)	100.01(11)
Crystal System	triclinic	triclinic
Space Group	*P*-1	*P*-1
a/Å	11.32210(10)	11.1612(2)
b/Å	18.08890(10)	18.0152(2)
c/Å	20.11190(10)	19.7961(3)
α/°	110.8450(10)	112.2100(10)
β/°	91.6360(10)	90.4110(10)
γ/°	107.8460(10)	107.6010(10)
V/Å^3	3620.08(5)	3479.14(9)
Z	4	4
Z'	2	2
Wavelength/Å	1.54184	1.54184
Radiation type	Cu K_λ	Cu K_λ
θ_{min}/°	2.379	2.434
θ_{max}/°	75.320	73.554
Measured Refl's.	60699	43979
Indep't Refl's	14716	13275
Refl's I≥2 σ(I)	13314	11202
R_{int}	0.0255	0.0662
Parameters	1154	930
Restraints	800	361
Largest Peak	0.866	1.265
Deepest Hole	-0.774	-0.871
GooF	1.049	1.091
wR_2 (all data)	0.1082	0.2514
wR_2	0.1061	0.2231
R_1 (all data)	0.0425	0.0846
R_1	0.0391	0.0702

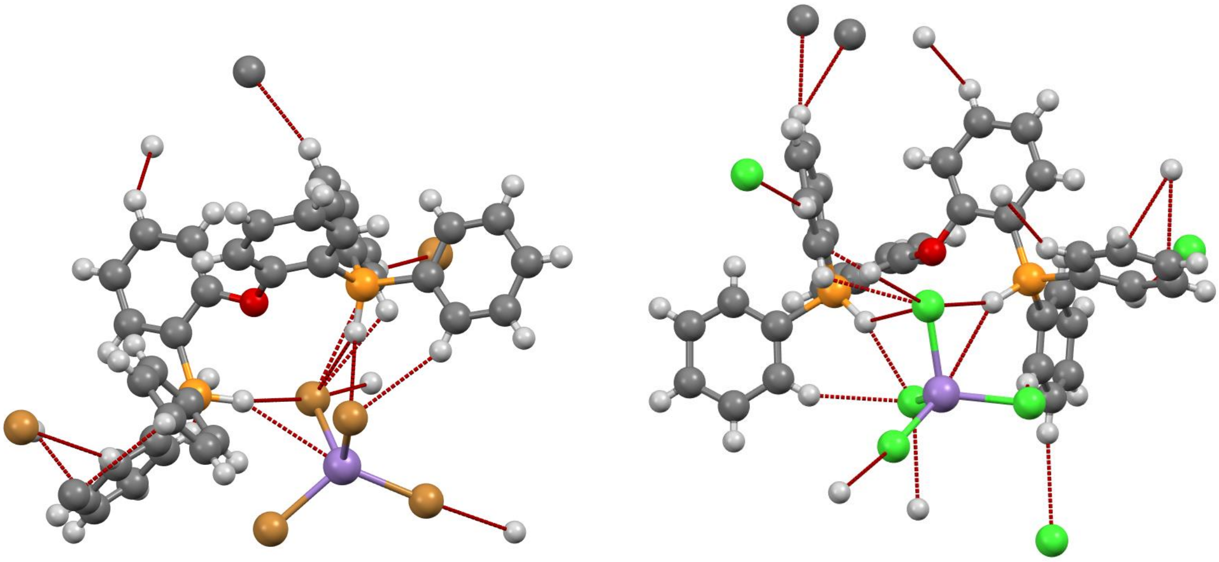

Fig. S1. Molecule structure of **1** (left) and **2** (right). Red dotted lines show short contacts.

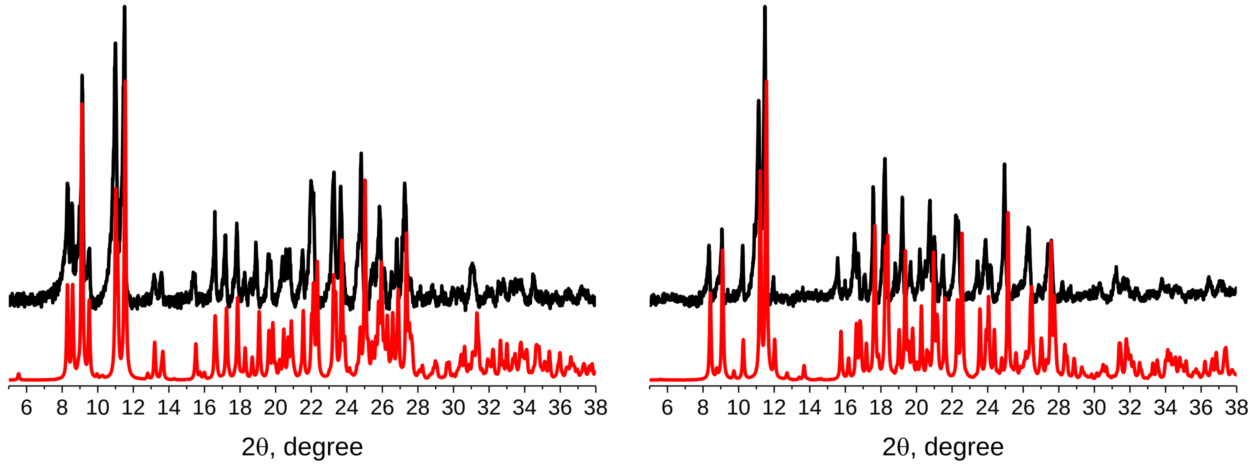

Fig. S2. Comparison of experimental (black) XRPD patterns of **1** (left) and **2** (right) with the diffraction pattern, simulated for the crystal structure (red).

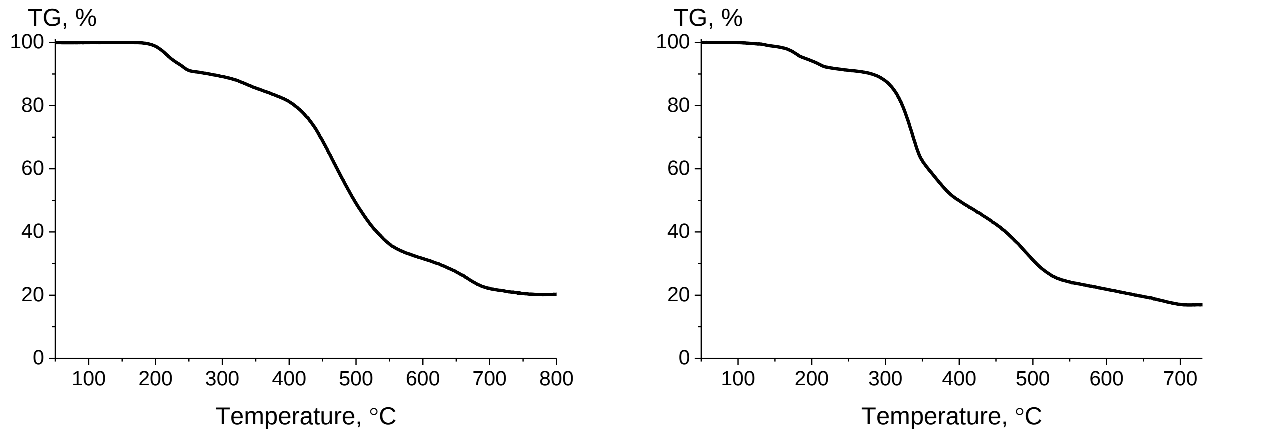

Fig. S3. TG curves of **1** (left) and **2** (right).

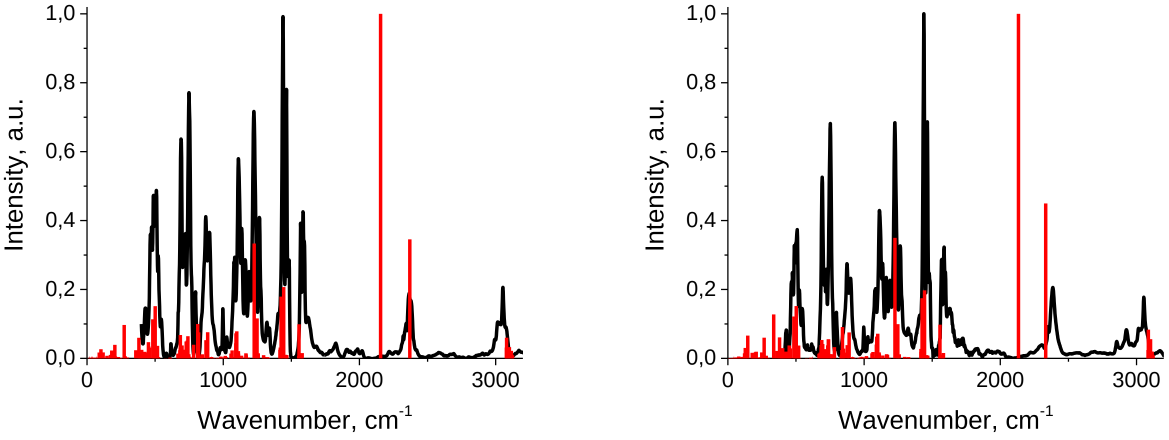

Fig. S4. Experimental (black) IR spectra of **1** (left) and **2** (right) with the calculated IR vibrations (red).

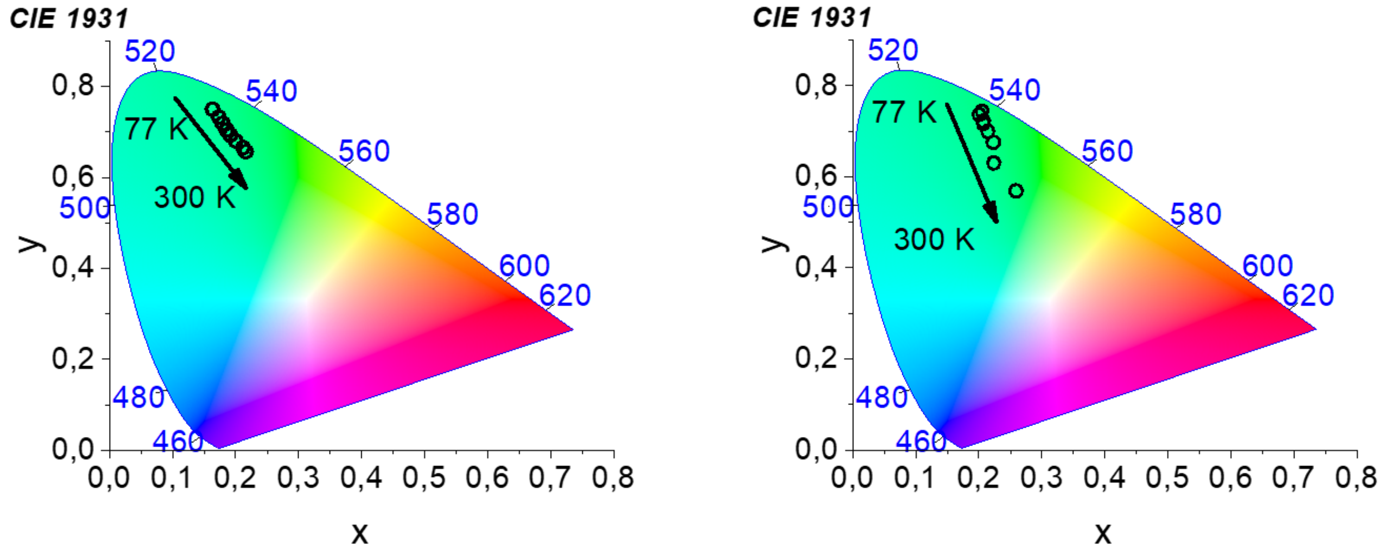

Fig. S5. Temperature dependences of the photoluminescence chromaticity of **1** (λ_{Ex} = 453 nm) and **2** (λ_{Ex} = 447 nm).

Table S2. Fractional Atomic Coordinates (×10^4^) and Equivalent Isotropic Displacement Parameters (Å^2×10^3) for **1**. U_{eq} is defined as 1/3 of the trace of the orthogonalised U_{ij}.

**Atom**	**x**	**y**	**z**	U_{eq}
Br8	12368(2)	6570.6(14)	440.4(11)	25.6(2)
Br7	13051.8(14)	8190.9(7)	-529.9(7)	26.9(2)
Br2	7376.9(14)	8189.0(9)	4571.6(9)	29.0(2)
Br5	14306(2)	8984.0(9)	1627.3(10)	30.4(2)
Br3	5265.7(13)	5808.6(9)	3384.7(7)	33.0(2)
Br1	6475.5(13)	6563.9(7)	5548.7(5)	33.3(2)
Br6	10474.4(14)	8304.5(9)	887.9(6)	35.6(2)
Br4	9056.3(13)	6383.6(9)	4175.5(4)	45.2(2)
Mn2	12490.0(18)	8065.0(12)	646.1(9)	21.2(3)
Mn1	7071.4(12)	6671.4(8)	4383.7(4)	24.5(2)
P3	5974.2(7)	7506.5(5)	340.9(4)	21.95(16)
P2	3883.4(8)	7519.4(5)	4687.8(4)	23.28(16)
P4	1962.8(7)	5990.0(5)	-1635.6(4)	22.76(16)
P1	7832.1(8)	8899.1(5)	6718.7(4)	25.54(17)
O2	4533(2)	6404.6(15)	-1066.9(11)	26.2(5)
O1	5323(2)	8632.1(15)	6103.6(11)	27.3(5)
C43	6846(3)	8294(2)	32.4(17)	27.5(5)
C56	3267(3)	6290.9(19)	-2075.6(16)	22.8(6)
C20	6481(3)	8774(2)	7162.6(17)	26.9(7)

Atom	x	y	z	U_{eq}
C7	2951(3)	6798(2)	5045.7(18)	30.1(5)
C13	3912(3)	8566(2)	5181.3(17)	25.5(6)
C49	6072(3)	6504.3(19)	-192.5(17)	24.4(6)
C67	1674(3)	4940(2)	-1687.5(16)	23.8(6)
C50	5368(3)	6063(2)	-881.1(17)	23.7(6)
C14	4596(3)	9013(2)	5873.4(17)	25.3(6)
C37	6555(3)	7770(2)	1259.5(16)	24.3(6)
C55	4453(3)	6429(2)	-1747.3(16)	23.7(6)
C1	3263(3)	7228(2)	3770.1(16)	24.5(6)
C25	8353(3)	9894(2)	6637.1(18)	28.3(5)
C68	2026(3)	4382(2)	-2251.6(19)	30.5(7)
C19	5339(3)	8674(2)	6808.1(17)	27.5(7)
C44	7901(11)	8265(6)	-250(5)	28.8(6)
C48	6446(10)	8997(7)	172(6)	27.8(6)
C51	5479(3)	5313(2)	-1335.7(18)	30.7(7)
C57	3139(3)	6348(2)	-2745.8(18)	32.4(8)
C21	6539(4)	8778(2)	7858.8(19)	38.1(9)
C32	9720(4)	9470(3)	7865(2)	38.3(5)
C58	4173(4)	6513(2)	-3080.9(19)	37.8(9)
C72	1001(3)	4673(2)	-1195.4(18)	31.9(7)
C59	5344(3)	6646(2)	-2750.1(19)	35.2(8)
C8	3087(8)	6034(5)	4910(4)	30.3(6)
C54	6887(4)	6176(2)	44(2)	41.2(9)
C18	3231(4)	8939(2)	4893(2)	39.6(9)
C15	4569(3)	9803(2)	6288.9(19)	34.3(8)
C60	5501(3)	6615(2)	-2076.1(19)	31.9(7)
C26	8064(7)	10563(5)	7037(4)	29.2(6)
C61	562(3)	6030(2)	-2032(2)	38.8(6)
C12	1979(9)	7005(5)	5432(5)	31.3(6)
C52	6309(4)	5008(2)	-1094(2)	40.2(9)
C46	8200(11)	9601(6)	-347(5)	28.2(7)
C38	5952(4)	7261(3)	1608(2)	38.8(9)
C2	2049(4)	6694(2)	3493(2)	41.5(9)
C47	7125(11)	9635(5)	-30(4)	27.8(6)
C24	4270(3)	8584(2)	7130.3(19)	34.9(8)
C4	2307(4)	6825(2)	2358.4(19)	37.5(8)
C9	2301(10)	5458(4)	5159(4)	30.8(6)
C33	10613(7)	9423(4)	8287(4)	39.7(6)
C71	710(3)	3831(2)	-1277(2)	36.9(9)
C42	7573(4)	8484(2)	1626.9(19)	39.4(9)
C10	1359(8)	5651(6)	5522(4)	31.1(7)
C5	3491(4)	7368(3)	2636(2)	45.5(10)
C31	9064(4)	8806(3)	7228(2)	39.4(5)
C39	6369(4)	7471(3)	2327(2)	41.3(9)
C23	4345(4)	8600(3)	7827(2)	43.4(10)
C70	1059(4)	3280(2)	-1836(2)	39.8(9)
C45	8594(8)	8919(7)	-450(5)	29.1(6)
C22	5467(4)	8696(3)	8186(2)	44.7(10)
C40	7378(4)	8184(3)	2694.1(19)	40.5(9)
C69	1692(4)	3545(2)	-2326(2)	41.1(9)
C17	3206(4)	9730(3)	5305(2)	49.7(11)
C53	7004(4)	5430(3)	-408(2)	47.6(11)
C6	3986(4)	7568(3)	3339(2)	43.3(10)
C41	7973(5)	8688(3)	2349(2)	49.9(11)
C11	1191(6)	6419(6)	5652(4)	31.5(7)
C3	1567(4)	6494(3)	2784(2)	49.3(11)
C30	9168(6)	9911(4)	6119(4)	28.8(6)

Atom	x	y	z	U_{eq}
C16	3863(4)	10148(3)	5997(2)	45.2(10)
C63	-1172(6)	5477(4)	-2988(4)	40.3(6)
C28	9376(6)	11377(5)	6450(4)	29.6(6)
C29	9660(6)	10683(4)	6039(3)	29.5(6)
C27	8587(6)	11326(4)	6953(4)	29.8(6)
C34	10862(5)	8634(3)	8074(3)	40.7(6)
C66	-103(5)	6483(4)	-1557(4)	40.9(7)
C36	9291(5)	8038(4)	7021(3)	40.9(6)
C64	-1806(5)	5938(4)	-2550(3)	42.3(6)
C35	10190(5)	7960(4)	7446(3)	41.8(6)
C65	-1275(5)	6438(4)	-1827(3)	42.9(7)
Br4A	9415(5)	6631(4)	4131(3)	48.6(6)
Mn1A	7402(5)	6872(3)	4381(3)	29
Br2A	7572(7)	8367(4)	4576(4)	27.6(6)
Br1A	6841(5)	6729(3)	5535(3)	34.5(6)
Br3A	5585(5)	5949(4)	3401(4)	33.9(6)
Br5A	14624(6)	9117(7)	1617(5)	30.3(7)
Br6A	10797(5)	8513(4)	837(3)	38.0(6)
Mn2A	12740(20)	8249(7)	636(8)	21.1(7)
Br8A	12508(18)	6749(5)	444(7)	25.2(7)
Br7A	13244(12)	8360(6)	-550(6)	26.6(6)
Br8B	12610(20)	6670(30)	405(14)	25.2(7)
Br5B	14400(40)	9130(17)	1575(14)	30.3(7)
Mn2B	12850(30)	8180(30)	609(15)	21.1(7)
Br7B	13522(12)	8369(9)	-512(7)	26.9(7)
Br6B	10510(20)	8199(13)	725(10)	36.5(6)
C26A	8043(13)	10573(9)	7211(6)	29.0(6)
C27A	8442(10)	11381(7)	7216(7)	29.6(6)
C28A	9128(11)	11547(7)	6688(7)	29.8(6)
C29A	9441(10)	10973(9)	6176(6)	29.6(6)
C30A	9057(11)	10131(7)	6146(6)	29.1(6)
C44A	7917(18)	8133(10)	-333(9)	28.8(7)
C45A	8663(13)	8714(11)	-542(8)	29.0(7)
C46A	8435(14)	9447(11)	-431(7)	28.5(7)
C47A	7443(19)	9617(8)	-124(8)	28.0(6)
C48A	6620(16)	9017(13)	115(10)	27.9(6)
C12A	1960(20)	6858(12)	5340(11)	31.2(7)
C11A	1257(15)	6179(16)	5568(9)	31.3(7)
C10A	1640(20)	5524(11)	5419(10)	30.9(7)
C9A	2640(20)	5433(10)	5084(9)	30.6(7)
C8A	3358(19)	6048(14)	4887(11)	30.3(7)
C66A	129(14)	6718(10)	-1765(9)	41.6(7)
C65A	-925(13)	6740(9)	-2126(8)	42.4(7)
C64A	-1403(13)	6138(9)	-2847(8)	41.7(7)
C63A	-862(14)	5540(11)	-3149(9)	40.2(7)
C62	34(3)	5500(2)	-2743(2)	38.2(6)
C36A	9741(17)	8259(11)	6813(10)	41.0(6)
C35A	10741(17)	8224(11)	7240(10)	41.3(6)
C34A	11328(17)	8877(11)	7865(10)	41.0(6)
C33A	10730(20)	9269(14)	8217(14)	40.0(6)

Table S3. Fractional Atomic Coordinates (×10^4) and Equivalent Isotropic Displacement Parameters (Å^2×10^3) for **2**. U_{eq} is defined as 1/3 of the trace of the orthogonalised U_{ij}.

Atom	x	y	z	U_{eq}
Mn01	5631.0(5)	-1849.6(4)	652.3(3)	28.24(19)

Atom	x	y	z	U_{eq}
Mn02	10520.9(5)	3268.2(4)	5615.4(3)	29.73(19)
Cl03	4320.5(8)	-3274.5(5)	426.7(4)	29.3(2)
P1	8940.0(8)	1097.9(5)	3315.7(5)	22.7(2)
P4	5949.9(7)	3941.4(5)	1618.2(4)	21.0(2)
P3	8508.3(7)	2531.9(5)	-359.8(4)	21.2(2)
Cl2	9290.6(8)	1833.2(6)	5412.5(5)	30.9(2)
P2	6385.3(8)	2464.6(5)	5341.9(4)	21.8(2)
Cl5	4750.8(9)	-1009.7(6)	1598.7(5)	35.8(2)
Cl1	10020.3(9)	3347.4(6)	4480.3(5)	36.6(2)
Cl6	5190.4(9)	-1747.4(6)	-488.6(5)	34.4(2)
Cl4	9639.3(9)	4097.0(6)	6570.4(5)	37.6(2)
Cl7	7799.8(10)	-1639(2)	892.0(9)	45.1(6)
Cl3	12706.1(9)	3539.9(9)	5842.3(6)	51.2(3)
O2	8181(2)	3585.3(16)	1091.0(12)	24.6(5)
O1	6653(2)	1365.3(16)	3881.0(12)	24.2(5)
C54	9373(3)	3955(2)	935.8(18)	22.5(6)
C19	5523(3)	982(2)	4084.7(19)	23.2(7)
C25	6248(3)	3223(2)	5002.6(18)	25.3(7)
C61	7012(3)	3680(2)	2091.6(18)	22.7(6)
C43	8552(3)	1717(2)	-67.6(18)	25.5(7)
C55	6662(3)	4985(2)	1647.9(18)	23.2(6)
C67	4531(3)	3910(2)	2040(2)	34.6(6)
C49	9632(3)	3527(2)	230.0(18)	23.9(7)
C7	7703(3)	1219(2)	2831.2(19)	26.8(7)
C8	6636(3)	1321(2)	3164.5(19)	27.1(7)
C53	10233(3)	4694(2)	1437(2)	28.4(7)
C44	9698(4)	1753(3)	252(2)	35.5(8)
C31	6052(3)	2746(2)	6271.6(19)	24.6(7)
C62	8088(3)	3559(2)	1781.4(18)	24.1(7)
C20	5310(3)	1430(2)	4795.8(19)	25.5(7)
C26	5090(4)	3127(3)	4646(2)	33.9(8)
C37	8860(3)	2290(2)	-1282.5(19)	24.3(7)
C30	7301(4)	3959(2)	5151(2)	32.4(8)
C68	4595(3)	4492(2)	2757(2)	33.8(6)
C1	10277(3)	1162(3)	2816(2)	36.5(6)
C66	6846(3)	3622(2)	2767.3(19)	29.9(8)
C34	5533(3)	3148(3)	7707(2)	33.2(8)
C56	7540(3)	5597(2)	2239(2)	32.7(8)
C2	10204(3)	489(3)	2152(2)	34.3(6)
C48	7452(4)	1025(2)	-202(2)	31.8(8)
C32	5450(4)	3338(2)	6574(2)	34.5(8)
C50	10787(4)	3885(3)	19(2)	38.0(9)
C40	9312(3)	1906(3)	-2727(2)	32.6(8)
C60	6223(3)	5201(2)	1109(2)	30.7(8)
C12	7765(4)	1219(3)	2126(2)	36.5(9)
C24	4679(4)	202(3)	3624(2)	36.0(9)
C47	7510(4)	375(3)	6(2)	38.9(9)
C28	6037(5)	4483(3)	4569(2)	41.9(10)
C65	7752(4)	3468(3)	3134(2)	36.6(9)
C63	8984(4)	3382(3)	2126(2)	32.3(8)
C38	9169(4)	1569(3)	-1664(2)	39.0(9)
C58	7588(4)	6648(3)	1766(2)	39.7(10)
C64	8812(4)	3355(3)	2814(2)	37.6(9)
C21	4218(4)	1055(3)	5059(2)	40.2(10)
C46	8640(5)	411(3)	330(2)	42.0(10)
C29	7181(4)	4580(3)	4921(2)	39.5(10)
C10	5736(4)	1415(3)	2113(2)	42.6(10)

Atom	x	y	z	U_{eq}
C52	11383(4)	5028(3)	1214(2)	37.7(9)
C33	5217(4)	3538(3)	7293(2)	42.2(10)
C3	11121(9)	506(8)	1724(7)	36.2(6)
C69	3502(4)	4491(3)	3078(2)	39.1(6)
C27	4997(4)	3754(3)	4429(2)	39.5(9)
C45	9738(4)	1109(3)	457(2)	40.7(9)
C9	5657(4)	1434(3)	2823(2)	35.9(9)
C39	9395(5)	1387(3)	-2381(2)	45.1(10)
C11	6788(4)	1317(3)	1771(2)	44.2(10)
C41	9029(4)	2640(3)	-2340(2)	38.9(9)
C59	6718(4)	6042(3)	1172(2)	37.9(10)
C23	3593(4)	-142(3)	3898(3)	48.1(12)
C57	7995(4)	6429(3)	2304(2)	42.0(10)
C51	11659(4)	4632(3)	514(3)	45.6(11)
C42	8797(4)	2832(3)	-1617(2)	39.9(9)
C36	6361(4)	2338(3)	6675(3)	49.4(12)
C35	6081(4)	2524(3)	7389(3)	48.2(12)
C22	3371(4)	276(3)	4613(3)	50.9(12)
C6	11266(7)	1930(5)	3029(4)	38.0(7)
C72	3378(7)	3253(5)	1716(5)	37.8(7)
C4	12221(7)	1322(5)	1947(4)	37.3(6)
C70	2401(7)	3851(5)	2786(5)	39.3(7)
C5	12267(6)	1991(5)	2607(4)	38.6(7)
C71	2315(7)	3248(5)	2092(4)	39.0(7)
C6A	11472(10)	1780(7)	3177(6)	38.1(7)
C5A	12505(9)	1767(7)	2795(6)	38.3(7)
C4A	12378(10)	1131(7)	2110(6)	37.7(6)
C3A	11324(12)	600(10)	1788(10)	36.3(6)
C72A	3394(9)	3458(6)	1556(6)	37.5(7)
C71A	2268(8)	3496(6)	1837(5)	38.9(7)
C70A	2304(9)	4050(6)	2565(6)	39.2(7)
C13	8464(3)	106(2)	3399.9(19)	25.3(5)
C14	7470(7)	-575(5)	3003(4)	26.0(6)
C18	9315(6)	92(5)	3937(4)	25.5(6)
C16	9071(7)	-667(4)	4035(3)	25.9(6)
C15	7259(6)	-1342(4)	3103(4)	26.5(6)
C17	8053(7)	-1371(5)	3607(4)	25.9(6)
C16A	8540(11)	-911(7)	3913(5)	26.1(6)
C18A	8970(9)	-76(7)	3922(6)	25.5(6)
C17A	7649(9)	-1544(6)	3354(6)	26.2(6)
C15A	7134(9)	-1376(6)	2815(6)	26.4(6)
C14A	7507(10)	-585(7)	2809(5)	25.9(6)
Cl7A	7809(14)	-1350(30)	831(15)	45.4(10)

Table S4. Experimental bond lengths in Å for **1**.

Atom	Atom	Length/Å
Br8	Mn2	2.544(3)
Br7	Mn2	2.5359(19)
Br2	Mn1	2.5438(13)
Br5	Mn2	2.495(3)
Br3	Mn1	2.4960(12)
Br1	Mn1	2.5134(11)
Br6	Mn2	2.480(3)
Br4	Mn1	2.4723(11)
P3	C43	1.786(3)

Atom	Atom	Length/Å
P3	C49	1.787(3)
P3	C37	1.784(3)
P2	C7	1.790(3)
P2	C13	1.788(3)
P2	C1	1.785(3)
P4	C56	1.784(3)
P4	C67	1.790(3)
P4	C61	1.788(4)
P1	C20	1.788(4)
P1	C25	1.783(3)
P1	C31	1.791(4)
O2	C50	1.385(4)
O2	C55	1.386(3)
O1	C14	1.386(4)
O1	C19	1.391(4)
C43	C44	1.344(13)
C43	C48	1.417(13)
C43	C44A	1.486(19)
C43	C48A	1.36(2)
C56	C55	1.389(4)
C56	C57	1.395(4)
C20	C19	1.388(5)
C20	C21	1.397(5)
C7	C8	1.369(10)
C7	C12	1.431(11)
C7	C12A	1.30(2)
C7	C8A	1.50(2)
C13	C14	1.395(4)
C13	C18	1.399(5)
C49	C50	1.397(4)
C49	C54	1.394(5)
C67	C68	1.386(5)
C67	C72	1.397(4)
C50	C51	1.386(5)
C14	C15	1.385(5)
C37	C38	1.381(5)
C37	C42	1.380(5)
C55	C60	1.384(5)
C1	C2	1.376(5)
C1	C6	1.382(5)
C25	C26	1.335(9)
C25	C30	1.413(8)
C25	C26A	1.497(14)
C25	C30A	1.391(13)
C68	C69	1.393(5)
C19	C24	1.381(5)
C44	C45	1.401(12)
C48	C47	1.377(10)
C51	C52	1.380(5)
C57	C58	1.374(5)
C21	C22	1.391(6)
C32	C33	1.344(8)
C32	C31	1.393(5)
C32	C33A	1.53(2)
C58	C59	1.378(5)
C72	C71	1.401(5)
C59	C60	1.385(5)

Atom	Atom	Length/Å
C8	C9	1.398(9)
C54	C53	1.382(5)
C18	C17	1.383(5)
C15	C16	1.383(6)
C26	C27	1.398(9)
C61	C66	1.419(7)
C61	C66A	1.411(15)
C61	C62	1.394(5)
C12	C11	1.375(10)
C52	C53	1.389(5)
C46	C47	1.398(8)
C46	C45	1.387(8)
C38	C39	1.384(5)
C2	C3	1.389(5)
C24	C23	1.391(5)
C4	C5	1.355(6)
C4	C3	1.376(6)
C9	C10	1.373(8)
C33	C34	1.455(8)
C71	C70	1.372(6)
C42	C41	1.389(5)
C10	C11	1.394(8)
C5	C6	1.382(5)
C31	C36	1.407(7)
C31	C36A	1.474(19)
C39	C40	1.372(6)
C23	C22	1.375(6)
C70	C69	1.371(6)
C40	C41	1.367(6)
C17	C16	1.382(6)
C30	C29	1.405(8)
C63	C64	1.366(8)
C63	C62	1.422(7)
C28	C29	1.374(9)
C28	C27	1.377(9)
C34	C35	1.394(7)
C66	C65	1.386(7)
C36	C35	1.383(7)
C64	C65	1.406(8)
Br4A	Mn1A	2.481(6)
Mn1A	Br2A	2.535(7)
Mn1A	Br1A	2.504(6)
Mn1A	Br3A	2.495(6)
Br5A	Mn2A	2.532(18)
Br6A	Mn2A	2.41(2)
Mn2A	Br8A	2.527(14)
Mn2A	Br7A	2.53(2)
Br8B	Mn2B	2.53(6)
Br5B	Mn2B	2.32(5)
Mn2B	Br7B	2.50(4)
Mn2B	Br6B	2.68(3)
C26A	C27A	1.387(16)
C27A	C28A	1.396(14)
C28A	C29A	1.323(15)
C29A	C30A	1.428(15)
C44A	C45A	1.34(2)
C45A	C46A	1.370(14)

Atom	Atom	Length/Å
C46A	C47A	1.361(14)
C47A	C48A	1.433(17)
C12A	C11A	1.48(3)
C11A	C10A	1.32(2)
C10A	C9A	1.363(19)
C9A	C8A	1.35(2)
C66A	C65A	1.398(19)
C65A	C64A	1.432(19)
C64A	C63A	1.37(2)
C63A	C62	1.320(16)
C36A	C35A	1.43(2)
C35A	C34A	1.35(2)
C34A	C33A	1.19(3)

Table S5. Experimental bond lengths in Å for **2**.

Atom	Atom	Length/Å
Mn01	Cl03	2.4045(10)
Mn01	Cl5	2.3551(10)
Mn01	Cl6	2.3939(10)
Mn01	Cl7	2.3497(14)
Mn01	Cl7A	2.295(15)
Mn02	Cl2	2.4062(10)
Mn02	Cl1	2.3778(10)
Mn02	Cl4	2.3565(10)
Mn02	Cl3	2.3422(11)
P1	C7	1.787(4)
P1	C1	1.794(4)
P1	C13	1.776(4)
P4	C61	1.778(3)
P4	C55	1.784(3)
P4	C67	1.792(3)
P3	C43	1.783(4)
P3	C49	1.785(4)
P3	C37	1.787(3)
P2	C25	1.778(4)
P2	C31	1.789(3)
P2	C20	1.786(4)
O2	C54	1.390(4)
O2	C62	1.389(4)
O1	C19	1.386(4)
O1	C8	1.390(4)
C54	C49	1.399(4)
C54	C53	1.375(5)
C19	C20	1.397(5)
C19	C24	1.382(5)
C25	C26	1.399(5)
C25	C30	1.408(5)
C61	C62	1.390(4)
C61	C66	1.390(4)
C43	C44	1.396(5)
C43	C48	1.397(5)
C55	C56	1.379(5)
C55	C60	1.397(5)
C67	C68	1.395(5)
C67	C72	1.400(8)

Atom	Atom	Length/Å
C67	C72A	1.399(11)
C49	C50	1.400(5)
C7	C8	1.393(5)
C7	C12	1.399(5)
C8	C9	1.385(5)
C53	C52	1.395(5)
C44	C45	1.377(6)
C31	C32	1.380(5)
C31	C36	1.374(5)
C62	C63	1.385(5)
C20	C21	1.411(5)
C26	C27	1.382(6)
C37	C38	1.382(5)
C37	C42	1.389(5)
C30	C29	1.395(6)
C68	C69	1.380(5)
C1	C2	1.393(5)
C1	C6	1.392(8)
C1	C6A	1.420(11)
C66	C65	1.392(5)
C34	C33	1.368(6)
C34	C35	1.385(6)
C56	C57	1.383(5)
C2	C3	1.334(12)
C2	C3A	1.447(15)
C48	C47	1.398(5)
C32	C33	1.378(5)
C50	C51	1.385(6)
C40	C39	1.373(6)
C40	C41	1.388(6)
C60	C59	1.402(5)
C12	C11	1.383(5)
C24	C23	1.399(6)
C47	C46	1.383(6)
C28	C29	1.381(7)
C28	C27	1.395(6)
C65	C64	1.379(5)
C63	C64	1.394(5)
C38	C39	1.375(5)
C58	C59	1.375(6)
C58	C57	1.386(6)
C21	C22	1.374(6)
C46	C45	1.404(6)
C10	C9	1.397(5)
C10	C11	1.384(6)
C52	C51	1.384(6)
C3	C4	1.508(12)
C69	C70	1.343(8)
C69	C70A	1.476(11)
C41	C42	1.385(6)
C23	C22	1.395(6)
C36	C35	1.385(6)
C6	C5	1.404(9)
C72	C71	1.405(10)
C4	C5	1.390(10)
C70	C71	1.371(10)
C6A	C5A	1.384(13)

Atom	Atom	Length/Å
C5A	C4A	1.377(14)
C4A	C3A	1.246(16)
C72A	C71A	1.388(11)
C71A	C70A	1.401(12)
C13	C14	1.338(9)
C13	C18	1.433(8)
C13	C18A	1.362(11)
C13	C14A	1.461(12)
C14	C15	1.419(10)
C18	C16	1.399(8)
C16	C17	1.385(8)
C15	C17	1.357(9)
C16A	C18A	1.426(12)
C16A	C17A	1.364(13)
C17A	C15A	1.378(12)
C15A	C14A	1.364(14)

Table S6. Cartesian coordinates of the **1**.

	x, Å	y, Å	z, Å
Br	10.335	10.974	0.818
Br	10.267	14.512	-0.984
Br	11.124	14.216	3.022
Br	7.205	13.615	1.649
Mn	9.633	13.389	1.2
Br	3.55	10.579	8.489
Br	2.548	7.394	6.285
Br	3.374	7.028	10.304
Br	6.475	7.775	7.754
Mn	4.056	8.11	8.14
P	2.583	12.662	0.633
P	-1.004	11.574	-3.037
O	1.643	11.85	-1.981
C	3.151	14.256	0.06
C	0.331	12.431	-3.854
C	3.28	11.348	-0.357
C	-0.746	9.806	-3.134
C	2.767	11.118	-1.636
C	3.042	12.409	2.339
C	1.578	12.416	-3.245
C	-0.006	9.28	-4.181
H	0.433	9.854	-4.796
C	4.378	14.424	-0.464
H	4.95	13.676	-0.584
C	2.301	15.359	0.319
H	1.458	15.236	0.742
C	3.335	10.177	-2.48
H	2.979	10.025	-3.348
C	0.193	13.045	-5.099
H	-0.661	13.09	-5.516

C	1.291	13.588	-5.721
H	1.199	13.983	-6.58
C	-1.389	8.967	-2.22
H	-1.908	9.327	-1.512
C	2.524	13.562	-5.107
H	3.276	13.941	-5.547
C	4.371	10.6	0.082
H	4.721	10.736	0.954
C	2.68	12.989	-3.855
H	3.527	12.99	-3.424
C	-2.59	11.948	-3.773
C	4.43	9.466	-2.031
H	4.84	8.83	-2.607
C	3.982	16.799	-0.644
H	4.263	17.667	-0.908
C	2.621	11.264	2.986
H	2.118	10.608	2.517
C	2.728	16.613	-0.056
H	2.161	17.363	0.086
C	-1.247	7.58	-2.371
H	-1.662	6.994	-1.75
C	3.778	13.355	3.021
H	4.078	14.14	2.578
C	2.936	11.071	4.321
H	2.647	10.284	4.766
C	-0.514	7.062	-3.409
H	-0.42	6.122	-3.498
C	4.812	15.704	-0.836
H	5.673	15.822	-1.219
C	3.662	12.016	5.003
H	3.876	11.881	5.918
C	0.084	7.896	-4.319
H	0.564	7.527	-5.052
C	4.943	9.664	-0.758
H	5.689	9.154	-0.461
C	4.076	13.15	4.362
H	4.573	13.804	4.838
C	-4.192	11.732	-5.549
H	-4.399	11.509	-6.449
C	-3.621	12.362	-2.891
H	-3.435	12.547	-1.979
C	-5.19	12.189	-4.735
H	-6.07	12.299	-5.075
C	-4.908	12.493	-3.393
H	-5.606	12.789	-2.821
C	-2.853	11.583	-5.094
H	-2.169	11.25	-5.662

H	1.197	12.623	0.576
H	-1.107	11.937	-1.838
P	-0.041	9.336	8.705
P	3.549	10.147	12.476
O	0.891	10.161	11.334
C	2.063	9.59	13.3
C	-0.717	7.818	9.37
C	-0.617	10.758	9.621
C	-0.13	10.995	10.906
C	-0.529	9.541	7.001
C	3.591	11.923	12.325
C	0.846	9.691	12.642
C	2.086	9.061	14.593
H	2.911	8.97	15.056
C	5.304	10.247	14.605
H	4.799	11.009	14.867
C	-0.132	6.607	9.118
H	0.68	6.57	8.626
C	-1.578	11.622	9.086
H	-1.894	11.493	8.2
C	-0.622	12.035	11.678
H	-0.298	12.183	12.559
C	2.87	12.767	13.067
H	2.243	12.432	13.698
C	-1.954	7.877	10.087
H	-2.365	8.712	10.278
C	-1.591	8.835	6.486
H	-2.055	8.209	7.03
C	-0.333	9.288	13.24
H	-1.157	9.355	12.773
C	-1.307	9.935	4.379
H	-1.558	10.051	3.47
C	-0.717	5.424	9.58
H	-0.289	4.589	9.429
C	6.317	9.841	15.388
H	6.548	10.318	16.177
C	-1.911	5.476	10.254
H	-2.316	4.673	10.557
C	-0.283	10.656	4.895
H	0.158	11.303	4.355
C	4.966	9.595	13.422
C	-0.297	8.779	14.534
H	-1.103	8.507	14.959
C	0.899	8.667	15.201
H	0.916	8.319	16.085
C	-2.069	12.667	9.851
H	-2.727	13.251	9.494

C	0.126	10.459	6.2
H	0.859	10.953	6.547
C	-2.534	6.699	10.495
H	-3.371	6.72	10.947
C	-1.986	9.037	5.17
H	-2.724	8.555	4.815
C	4.535	12.352	11.363
H	5.046	11.737	10.85
C	-1.596	12.854	11.136
H	-1.95	13.564	11.66
C	3.938	14.621	11.977
H	4.05	15.557	11.861
C	4.668	13.743	11.214
H	5.277	14.083	10.569
C	3.044	14.146	12.911
H	2.549	14.752	13.449
C	7.048	8.647	14.993
H	7.765	8.33	15.529
C	5.66	8.432	13.038
H	5.418	7.97	12.243
C	6.697	7.97	13.827
H	7.172	7.187	13.573
H	1.266	9.331	8.802
H	3.643	9.635	11.309
H	4.824	10.987	14.961
Br	6.747	8.235	7.671
Mn	4.32	8.458	8.135
Br	3.672	10.882	8.497
Br	3.697	7.323	10.278
Br	2.83	7.623	6.315
Br	11.411	14.453	3.003
Br	7.457	14.013	1.554
Mn	9.815	13.714	1.181
Br	10.395	11.279	0.824
Br	10.392	14.818	-1.021
Br	10.556	11.173	0.752
Br	11.152	14.507	2.925
Mn	9.979	13.616	1.131
Br	10.7	14.805	-0.951
Br	7.313	13.559	1.346
C	2.831	12.65	13.39
H	2.353	12.168	14.054
C	2.835	14.038	13.4
H	2.354	14.513	14.066
C	3.55	14.73	12.419
H	3.531	15.679	12.438
C	4.252	14.136	11.468

H	4.729	14.654	10.83
C	4.285	12.71	11.413
H	4.796	12.287	10.732
C	4.474	14.26	-0.618
H	4.935	13.444	-0.775
C	5.009	15.422	-1.006
H	5.86	15.433	-1.43
C	4.338	16.598	-0.8
H	4.753	17.411	-1.066
C	3.103	16.655	-0.23
H	2.656	17.486	-0.128
C	2.49	15.437	0.214
H	1.628	15.455	0.612
C	-1.889	7.695	9.916
H	-2.454	8.451	10.038
C	-2.322	6.35	10.339
H	-3.145	6.217	10.794
C	-1.517	5.337	10.063
H	-1.811	4.469	10.319
C	-0.315	5.438	9.441
H	0.193	4.654	9.263
C	0.169	6.649	9.075
H	1.015	6.75	8.655
C	-3.477	12.927	-3.277
H	-3.264	13.38	-2.47
C	-4.662	13.243	-3.948
H	-5.337	13.763	-3.527
C	-4.828	12.762	-5.287
H	-5.603	12.995	-5.783
C	-3.866	11.965	-5.847
H	-3.928	11.694	-6.756
H	-2.23	10.991	-5.497
C	6.059	8.973	12.651
H	6.019	8.843	11.711
C	7.186	8.583	13.444
H	7.747	7.874	13.155
C	7.453	9.226	14.605
H	8.349	9.356	14.892
C	6.539	9.63	15.258
H	6.582	9.565	16.205

Table S7. Cartesian coordinates of the **2**.

	x, Å	y, Å	z, Å
Mn	7.283	-3.691	1.184
Cl	6.6	-5.96	0.775
Cl	5.83	-2.996	2.902
Cl	6.752	-2.615	-0.887
Cl	9.586	-3.519	1.619

Mn	9.882	1.178	10.194
Cl	9.294	-1.126	9.825
Cl	9.297	2.211	8.133
Cl	8.433	1.848	11.927
Cl	12.17	1.466	10.605
P	9.333	-0.733	6.019
P	5.708	0.014	9.697
O	6.627	-0.72	7.045
C	5.571	-1.539	7.415
C	5.147	1.585	9.081
C	7.893	-0.142	5.139
C	6.642	-0.23	5.744
C	5.17	-0.236	11.385
C	5.079	-1.331	8.706
C	3.912	1.701	8.434
H	3.383	0.929	8.268
C	5.919	2.731	9.351
H	6.747	2.658	9.809
C	10.797	-0.228	5.112
C	4.351	-0.679	13.99
H	4.089	-0.807	14.894
C	11.092	-0.859	3.906
H	10.543	-1.582	3.626
C	4.171	0.541	11.934
H	3.751	1.221	11.418
C	7.972	0.415	3.859
H	8.816	0.486	3.427
C	5.061	-2.514	6.579
H	5.397	-2.633	5.698
C	4.231	4.091	8.294
H	3.911	4.944	8.027
C	4.061	-2.183	9.184
H	3.73	-2.075	10.066
C	5.45	3.979	8.933
H	5.973	4.756	9.091
C	5.601	0.761	3.836
H	4.82	1.052	3.381
C	3.792	0.317	13.239
H	3.124	0.871	13.628
C	12.112	-0.492	3.13
H	12.339	-0.985	2.349
C	3.469	2.949	8.04
H	2.636	3.031	7.591
C	5.493	0.234	5.125
H	4.652	0.193	5.566
C	6.834	0.863	3.215
H	6.897	1.242	2.346

C	4.032	-3.322	7.076
H	3.653	-3.987	6.514
C	5.731	-1.255	12.117
H	6.413	-1.794	11.733
C	5.307	-1.5	13.413
H	5.67	-2.227	13.904
C	3.547	-3.168	8.374
H	2.864	-3.744	8.699
C	11.48	0.923	5.498
H	11.21	1.405	6.272
C	12.892	0.733	3.534
H	13.589	1.069	2.983
C	12.57	1.361	4.732
H	13.094	2.093	5.035
C	9.341	-2.502	6.172
C	8.608	-3.358	5.451
H	7.972	-3.036	4.823
C	10.291	-2.95	7.147
H	10.807	-2.334	7.652
C	10.43	-4.331	7.325
H	11.034	-4.669	7.975
C	8.789	-4.754	5.633
H	8.286	-5.373	5.115
C	9.684	-5.202	6.548
H	9.802	-6.138	6.661
H	9.46	-0.114	7.17
H	7.1	-0.026	9.694
P	4.471	5.49	2.937
P	8.122	4.632	-0.653
O	7.162	5.295	1.98
C	8.294	6.053	1.699
C	5.792	4.668	3.797
C	8.611	3.002	-0.123
C	4.697	7.259	2.991
C	2.898	5.103	3.703
C	8.826	5.875	0.418
C	8.844	6.926	2.609
H	8.484	7.015	3.482
C	9.866	2.811	0.457
H	10.447	3.55	0.593
C	7.063	4.705	3.234
C	8.66	4.945	-2.328
C	2.642	5.537	5.005
H	3.311	6.009	5.488
C	5.629	4.035	5.023
H	4.764	3.98	5.412
C	5.335	7.843	4.064

H	5.741	7.296	4.727
C	7.762	1.92	-0.367
H	6.917	2.05	-0.781
C	9.923	6.656	0.034
H	10.282	6.576	-0.842
C	9.394	5.426	-4.95
H	9.63	5.576	-5.858
C	4.097	8.055	2.013
H	3.634	7.66	1.283
C	8.178	0.639	0.011
H	7.607	-0.105	-0.139
C	6.718	3.481	5.689
H	6.602	3.067	6.536
C	8.155	4.129	3.859
H	9.011	4.134	3.448
C	9.403	4.008	-3.021
H	9.662	3.196	-2.6
C	4.823	10.021	3.206
H	4.872	10.968	3.279
C	7.968	3.539	5.108
H	8.714	3.171	5.568
C	9.415	0.445	0.599
H	9.693	-0.428	0.847
C	9.949	7.675	2.204
H	10.344	8.287	2.815
C	1.418	5.282	5.587
H	1.189	5.734	6.392
C	10.258	1.544	0.83
H	11.102	1.413	1.246
C	9.764	4.262	-4.322
H	10.279	3.62	-4.797
C	8.673	6.381	-4.248
H	8.434	7.2	-4.666
C	4.19	9.45	2.128
H	3.813	10.003	1.454
C	5.388	9.221	4.182
H	5.813	9.616	4.935
C	10.482	7.548	0.933
H	11.232	8.072	0.679
C	8.299	6.14	-2.935
H	7.798	6.789	-2.454
C	1.974	4.231	3.115
H	2.15	3.849	2.263
C	0.542	4.413	5.057
H	-0.229	4.145	5.542
C	0.785	3.926	3.798
H	0.136	3.37	3.381

H	4.363	5.168	1.779
H	6.675	4.697	-0.654
H	10.55	-1.551	3.546
H	1.286	5.33	6.528
C	11.789	0.548	5.767
H	11.657	0.868	6.651
C	12.955	0.827	5.074
H	13.609	1.399	5.458
C	13.169	0.276	3.83
H	14.012	0.4	3.408
C	12.287	-0.381	3.246
H	12.405	-0.579	2.323
C	1.882	4.709	2.825
H	2.064	4.555	1.905
C	0.601	4.553	3.335
H	-0.088	4.19	2.791
C	0.329	4.929	4.656
H	-0.571	4.958	4.958
C	9.972	-4.654	7.103
H	10.399	-5.148	7.793
C	9.997	-3.227	7.12
H	10.48	-2.776	7.801
C	9.331	-5.299	6.088
H	9.337	-6.248	6.056
C	8.672	-4.585	5.11
H	8.216	-5.058	4.422
C	8.658	-3.222	5.099
H	8.21	-2.749	4.408
Cl	9.439	-2.974	1.509

Table S8. Cartesian coordinates of the DFT optimized structure **1**.

	x, Å	y, Å	z, Å
P	0.078	9.187	8.644
P	3.52	10.157	12.327
O	0.918	10.032	11.231
Mn	3.938	7.873	8.719
Br	3.507	10.461	8.684
Br	2.389	7.015	6.989
Br	2.815	7.178	10.882
Br	6.332	7.538	8.903
H	3.131	8.761	14.94
H	4.908	10.938	14.768
H	0.64	6.375	9.074
H	-1.562	11.525	7.783
H	-0.01	12.24	12.481
H	2.219	11.836	14.378
H	-2.516	9.201	9.966
H	-2.273	8.471	7.076
H	-1.27	9.256	12.614
H	-1.126	9.852	3.151
H	-0.641	4.592	10.258

H	7.024	10.118	15.799
H	-2.863	5.102	11.264
H	1.1	10.537	4.037
H	-1.166	8.279	14.924
H	1.027	8.012	16.072
H	-2.292	13.561	9.042
H	1.648	10.188	6.45
H	-3.812	7.402	11.107
H	-2.812	8.813	4.661
H	4.403	12.347	10.68
H	-1.531	13.907	11.389
H	2.988	15.8	12.852
H	4.188	14.818	10.901
H	2.006	14.312	14.595
H	8.371	8.367	14.645
H	5.481	8.19	11.452
H	7.598	7.429	12.457
H	1.465	8.989	8.836
H	3.52	9.953	10.907
C	2.095	9.456	13.181
C	-0.871	7.886	9.445
C	-0.414	10.742	9.431
C	0.013	10.96	10.75
C	-0.297	9.341	6.897
C	3.353	11.947	12.536
C	0.855	9.588	12.543
C	2.161	8.884	14.456
C	5.487	10.148	14.285
C	-0.333	6.59	9.519
C	-1.242	11.688	8.813
C	-0.382	12.088	11.467
C	2.664	12.495	13.63
C	-2.12	8.185	10.018
C	-1.549	8.942	6.409
C	-0.325	9.175	13.153
C	-0.895	9.712	4.209
C	-1.06	5.596	10.181
C	6.677	9.694	14.855
C	-2.305	5.886	10.749
C	0.356	10.102	4.705
C	5.052	9.592	13.068
C	-0.251	8.618	14.435
C	0.982	8.466	15.082
C	-1.647	12.824	9.521
C	0.667	9.917	6.053
C	-2.839	7.178	10.665
C	-1.845	9.13	5.055
C	3.904	12.782	11.549
C	-1.221	13.016	10.842
C	3.091	14.717	12.764
C	3.769	14.168	11.67
C	2.538	13.883	13.744
C	7.434	8.71	14.202
C	5.806	8.609	12.407
C	7.004	8.178	12.982

Table S9. Cartesian coordinates of the DFT optimized structure **2**.

	x, Å	y, Å	z, Å
P	9.317	-0.774	6.182
P	5.895	0.17	9.767
O	6.675	-0.668	7.174
Mn	9.728	1.395	9.712
Cl	9.25	-1.047	9.697
Cl	8.707	2.04	7.641
Cl	8.222	2.154	11.339
Cl	11.986	1.703	9.594
H	3.327	0.383	8.422
H	6.674	2.952	9.397
H	4.659	-0.587	15.238
H	10.751	-1.567	3.775
H	3.517	0.841	11.329
H	9.043	0.72	3.611
H	5.564	-2.74	5.838
H	3.283	4.525	7.224
H	4.031	-2.043	10.546
H	5.531	4.849	8.253
H	4.754	1.238	3.464
H	2.968	0.451	13.734
H	12.881	-0.754	2.773
H	2.172	2.294	7.322
H	4.546	0.186	5.731
H	6.994	1.523	2.415
H	3.892	-4.303	6.864
H	7.462	-0.823	11.955
H	6.902	-1.222	14.359
H	3.138	-3.965	9.214
H	11.276	1.188	7.092
H	14.215	0.998	3.942
H	13.413	1.942	6.115
H	8.245	-2.362	3.932
H	9.885	-3.027	7.878
H	9.623	-5.488	7.564
H	7.996	-4.824	3.618
H	8.68	-6.384	5.438
H	9.284	-0.625	7.61
H	7.302	0.199	9.595
C	5.685	-1.527	7.614
C	5.062	1.563	8.984
C	7.931	-0.023	5.301
C	6.665	-0.163	5.883
C	5.505	-0.004	11.508
C	5.263	-1.314	8.935
C	3.798	1.368	8.398
C	5.691	2.818	8.943
C	10.872	-0.22	5.476
C	4.895	-0.427	14.185
C	11.324	-0.778	4.266
C	4.244	0.37	11.994
C	8.053	0.591	4.05
C	5.201	-2.592	6.856
C	3.781	3.691	7.723
C	4.349	-2.202	9.515

C	5.04	3.876	8.303
C	5.645	0.878	3.979
C	3.941	0.155	13.341
C	12.523	-0.328	3.712
C	3.155	2.439	7.773
C	5.514	0.281	5.239
C	6.905	1.039	3.389
C	4.274	-3.46	7.442
C	6.473	-0.576	12.349
C	6.155	-0.789	13.692
C	3.852	-3.273	8.765
C	11.618	0.764	6.145
C	13.272	0.656	4.373
C	12.825	1.191	5.585
C	9.116	-2.554	5.907
C	8.567	-3.053	4.714
C	9.496	-3.429	6.939
C	9.336	-4.806	6.762
C	8.417	-4.432	4.546
C	8.802	-5.307	5.569

Table S10. Calculated zero-field splitting parameters.[a]

	**1**	**2**
D_{tot} [MHz]	4800	2007
E_{tot} [MHz]	525	356
D_{SS} [MHz][b]	443	481
E_{SS} [MHz][b]	52	63
D_{SOC} [MHz][c]	4368	1531
E_{SOC} [MHz][c]	485	296
$D_{\alpha\to\beta}$ [MHz][d]	8068	651
$E_{\alpha\to\beta}$ [MHz][d]	790	129
$D_{\alpha\to\alpha}/D_{\alpha\to\beta}$[d]	0.18	0.87
$E_{\alpha\to\alpha}/E_{\alpha\to\beta}$[d]	0.05	0.60
$D_{\beta\to\beta}/D_{\alpha\to\beta}$[d]	0.19	1.3
$E_{\beta\to\beta}/E_{\alpha\to\beta}$[d]	0.31	0.83
$D_{\beta\to\alpha}/D_{\alpha\to\beta}$[d]	-0.83	-0.85
$E_{\beta\to\alpha}/E_{\alpha\to\beta}$[d]	-0.61	-0.17

Table S11. Energy of transitions for polycrystalline complexes **1** and **2**.

	**1**	**2**
$^6A_1 \rightarrow {}^4T_1$ [cm^{-1}]	20700	20790
$^6A_1 \rightarrow {}^4T_2$ [cm^{-1}]	22230	22470
$^6A_1 \rightarrow {}^4A_1/{}^4E_1$ [cm^{-1}]	22730	23040
$\Delta(^4T_2 - {}^4T_1)$ [cm^{-1}]	1530	1680

Table S12. Calculated spin-flip transition energies of **1** and **2**.

**1**			**2**		
Functional	BP86	B3LYP		BP86	B3LYP
№	ΔE, cm^{-1}	ΔE, cm^{-1}	№	ΔE, cm^{-1}	ΔE, cm^{-1}
1	2789	903	1	2831	914
2	19194	19858	2	19867	20104
3	19505	19966	3	20299	20127
4	20060	20529	4	20869	20850
5	20269	20590	5	21201	20869
6	20439	20651	6	21255	20984
7	20516	20905	7	21379	21076
8	20599	21027	8	21487	21371
9	20854	21368	9	21588	21725
10	20975	21502	10	21678	21861
11	21005	21660	11	21727	21950
12	21170	21770	12	22111	22075
13	21903	21943	13	22697	22302
14	22609	22870	14	23347	23160
15	22622	22943	15	23378	23217
16	25504	27967	16	27139	28702
17	25961	28444	17	27495	29238
18	26123	28806	18	27840	29793
19	27912	29640	19	29632	30410
20	27935	30267	20	29864	31116
21	27983	30638	21	29907	31605
22	28232	31249	22	30086	32191
23	28239	31345	23	30171	32309
24	28393	31597	24	30312	32331
25	28714	31781	25	30630	32757

Table S13. Calculated UVVis transition energies of [H_2DPEphos][$ZnBr_4$] and [H_2DPEphos][$ZnCl_4$].

	[H_2DPEphos][$ZnBr_4$]								
Functional	BP86			B3LYP			CAM-B3LYP		
Unit	eV	cm^{-1}	nm	eV	cm^{-1}	nm	eV	cm^{-1}	nm
$\Delta E(\,^1\pi\pi)$	4.1329	33334	300.0	4.5849	36980	270.4	4.5949	37060	269.8
$\Delta E(\,^3\pi\pi)$	3.2007	25815	387.4	3.6347	29316	341.1	3.2327	26073	383.5
$\Delta E(\,^1\pi\pi - \,^3\pi\pi)$	0.9322	7519	-	0.9502	7664	-	1.3622	10987	-

	[H_2DPEphos][$ZnCl_4$]								
Functional	BP86			B3LYP			CAM-B3LYP		
Unit	eV	cm^{-1}	nm	eV	cm^{-1}	nm	eV	cm^{-1}	nm
$\Delta E(\,^1\pi\pi)$	4.3743	35281	283.4	4.4842	36168	276.5	4.4648	36011	277.7
$\Delta E(\,^3\pi\pi)$	3.3454	26982	370.6	3.4612	27916	358.2	3.3086	26686	374.7
$\Delta E(\,^1\pi\pi - \,^3\pi\pi)$	1.0289	8299	-	1.0230	8251	-	1.1562	9325	-

Table S14. Calculated UVVis transition energies of $[H_2DPEphos]^{2+}$ with different geometries.

	$[H_2DPEphos]^{2+}$					
Geometry from	$[H_2DPEphos][ZnBr_4]$			$[H_2DPEphos][ZnCl_4]$		
Functional	CAM-B3LYP			CAM-B3LYP		
Unit	eV	cm^{-1}	nm	eV	cm^{-1}	nm
$\Delta E(\,^1\pi\pi)$	4.6448	37463	266.9	4.6599	37585	266.1
$\Delta E(\,^3\pi\pi)$	3.2277	26033	384.1	3.2323	26070	383.6
$\Delta E(\,^1\pi\pi - \,^3\pi\pi)$	1.4171	11430	-	1.4276	11514	-

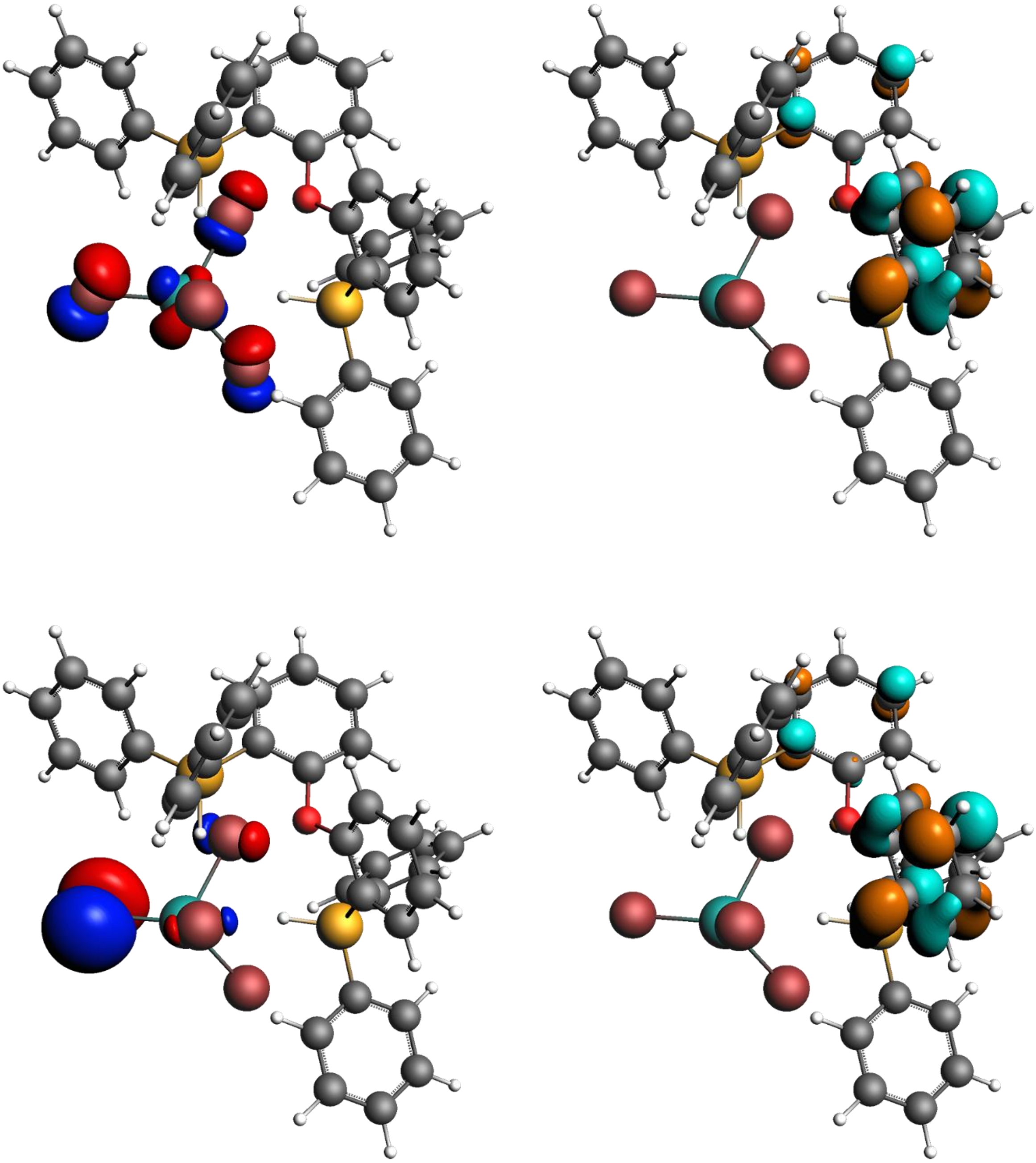

Fig. S6. The HOMO (left) and LUMO (right) of **1** for the spin-up (top) and spin-down (bottom) polarizations.

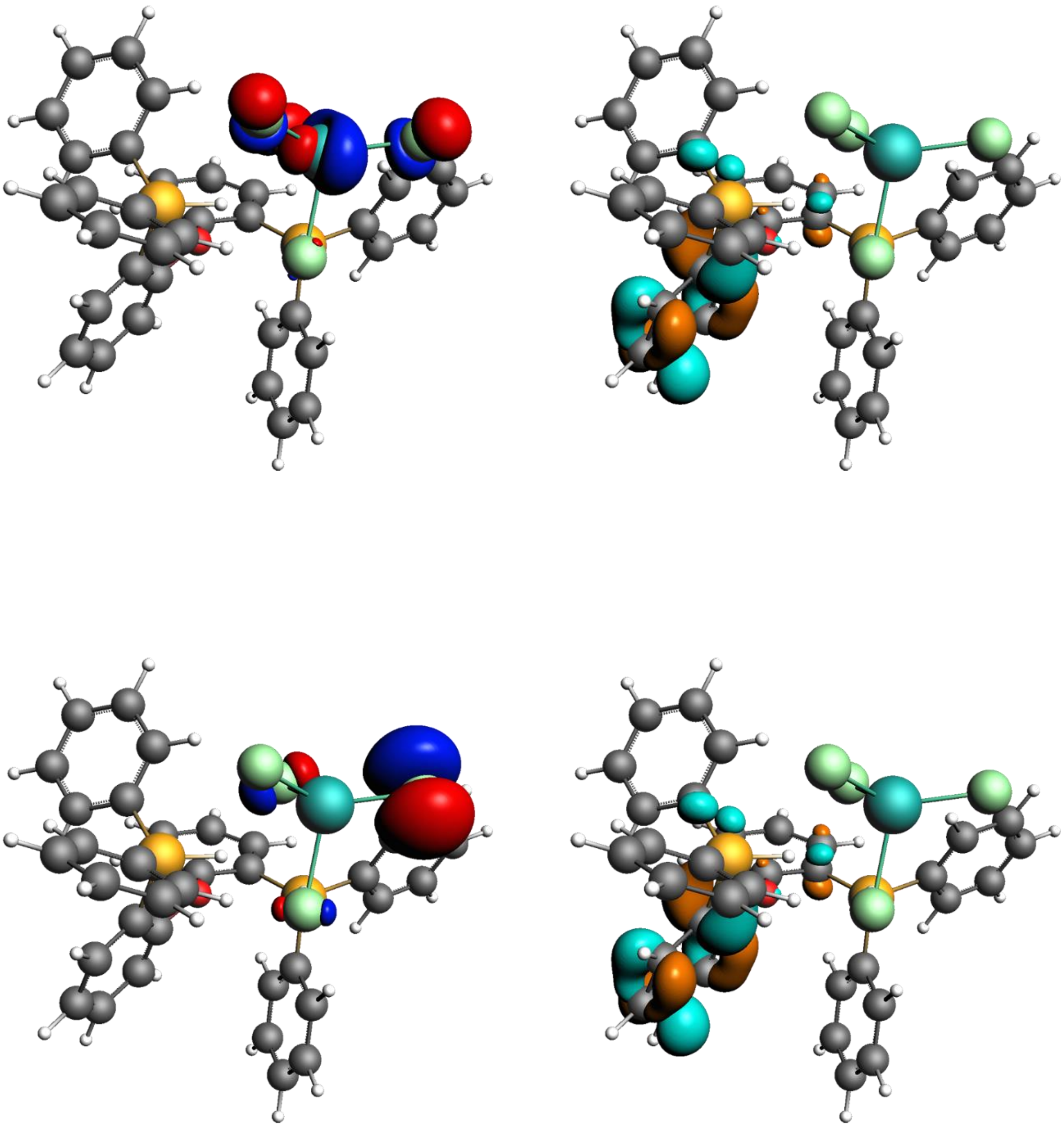

Fig. S7. The HOMO (left) and LUMO (right) of **2** for the spin-up (top) and spin-down (bottom) polarizations.

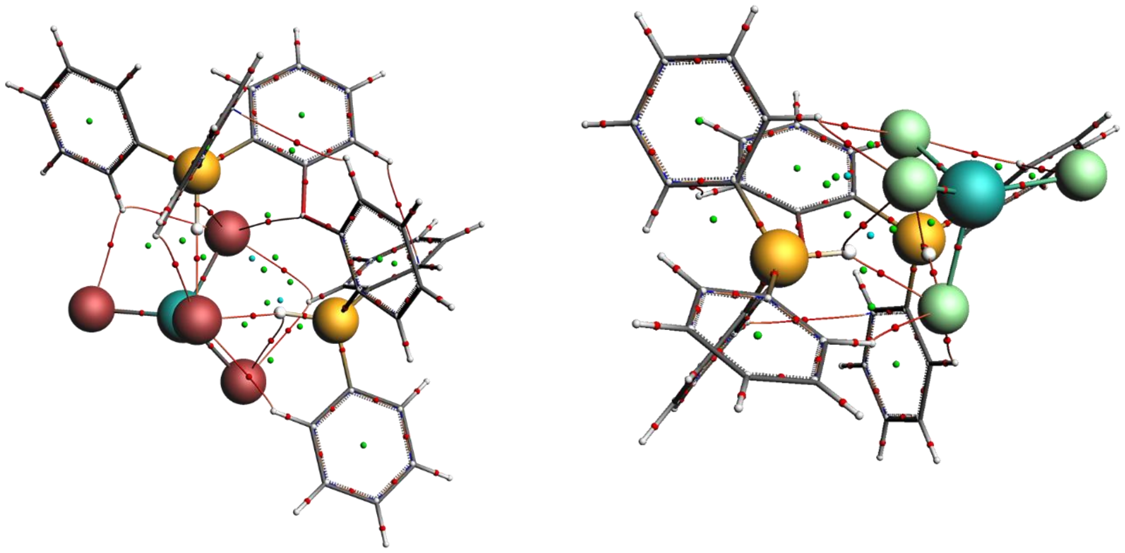

Fig. S8. The QTAIM (quantum theory of atoms in molecules) for **1** (left) and **2** (right). Red dot – (3, -1) bond critical points, green dot – (3, +1) ring critical points, turquoise dot – (3, +3) cage critical points, white dot – (3, -3) atom critical points.

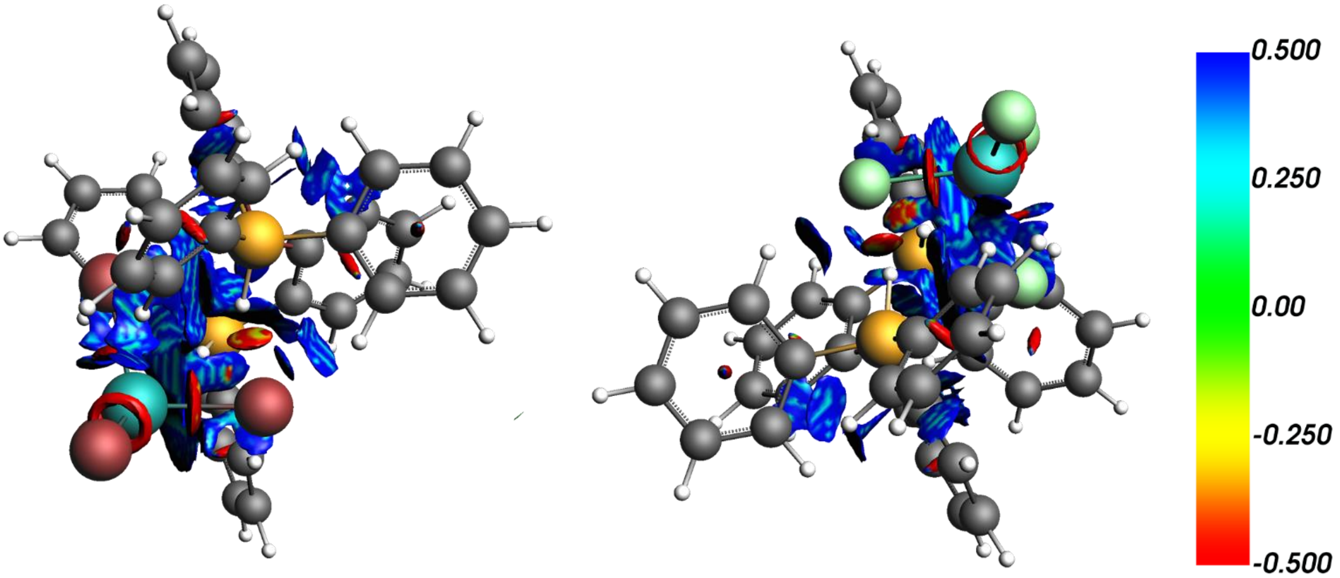

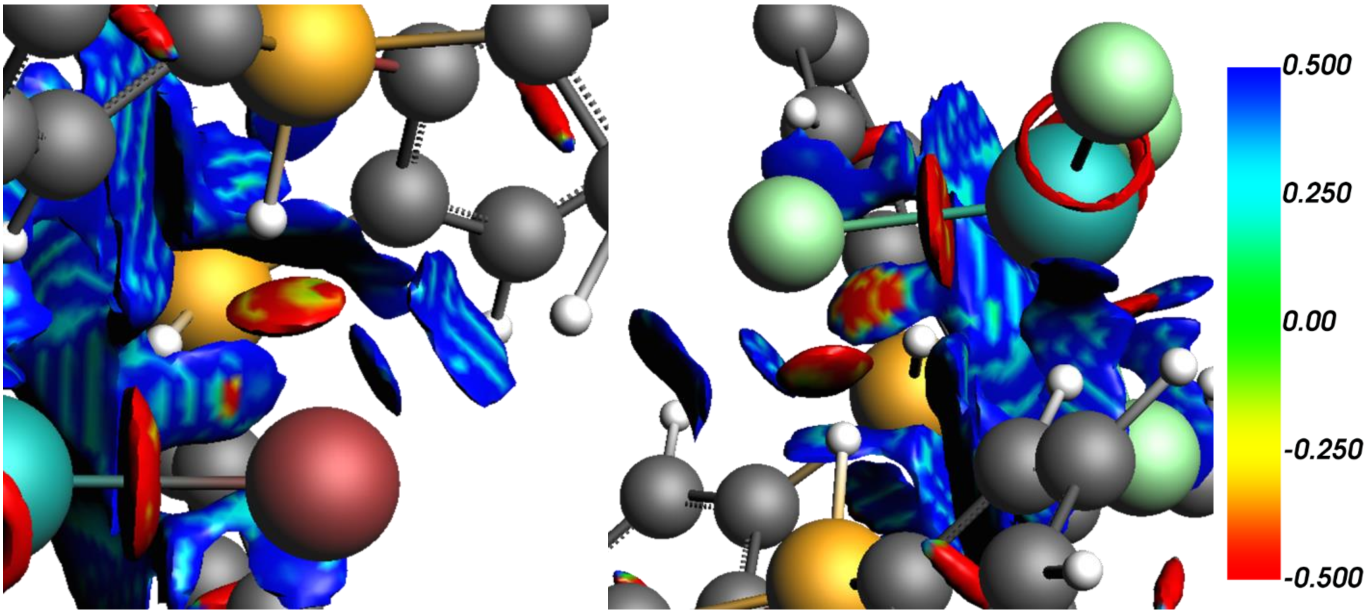

Fig. S9. The noncovalent interaction (NCI) plots for **1** (left) and **2** (right) showing PH…X(Mn) interaction. NCIs (SCF density) are presented by isosurfaces (s = 0.5 a.u., ρ= 0.02 a.u.) colored according to sign(λ_2)ρ in the red-green-blue scheme.

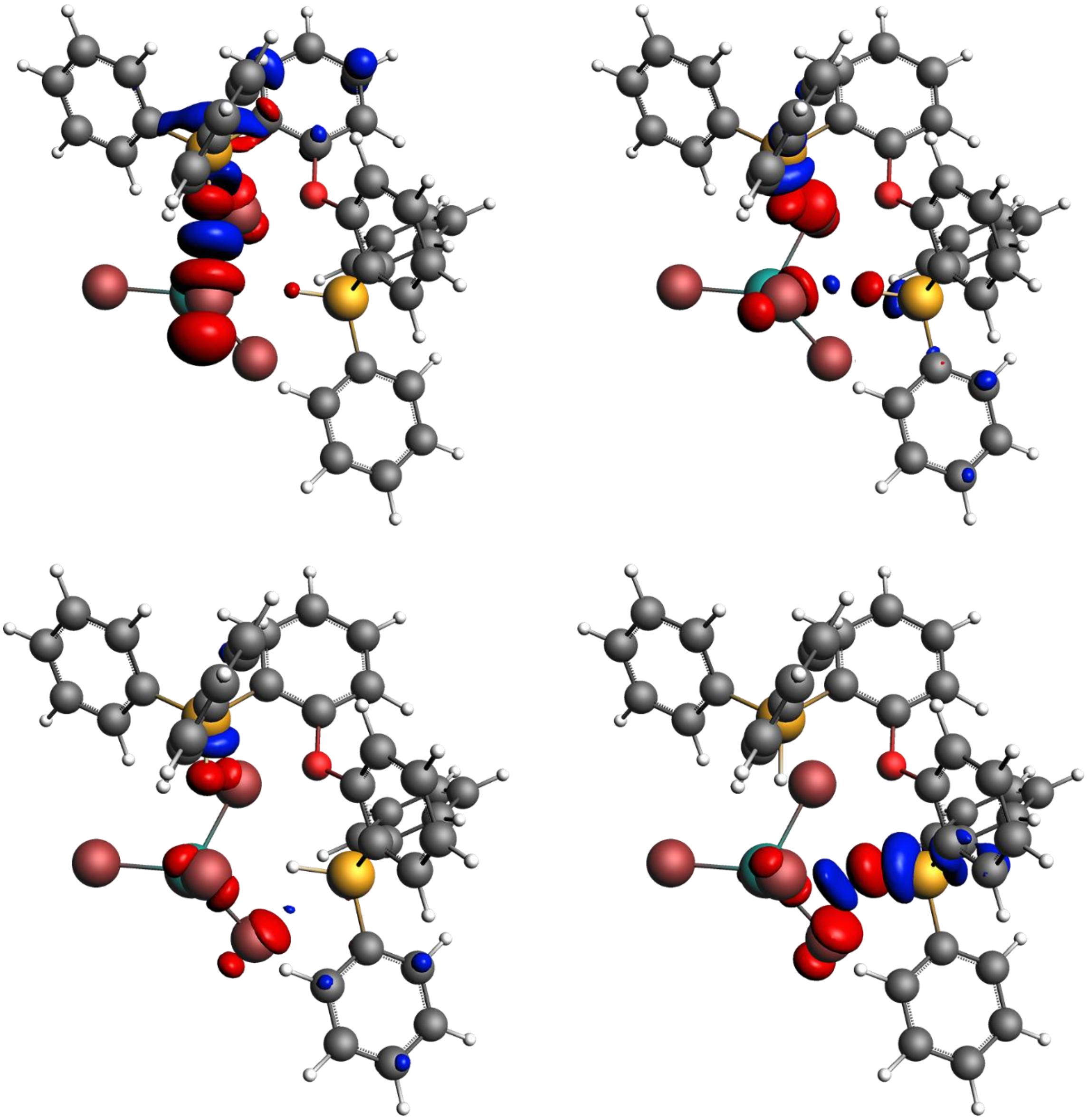

Fig. S10. The calculated deformation density $\Delta\rho_i$ for the $[MnBr_4]^{2-}$ anion with $[H_2DPEphos]^{2+}$ cation (**1**) with NOCV eigenvalues $|V| > 0.1$: left-top – $|V| = 0.1904$; right-top – $|V| = 0.1520$; left-bottom – $|V| = 0.1401$; right-bottom – $|V| = 0.1326$. Red for $\Delta\rho_i < 0$ and blue for $\Delta\rho_i > 0$.

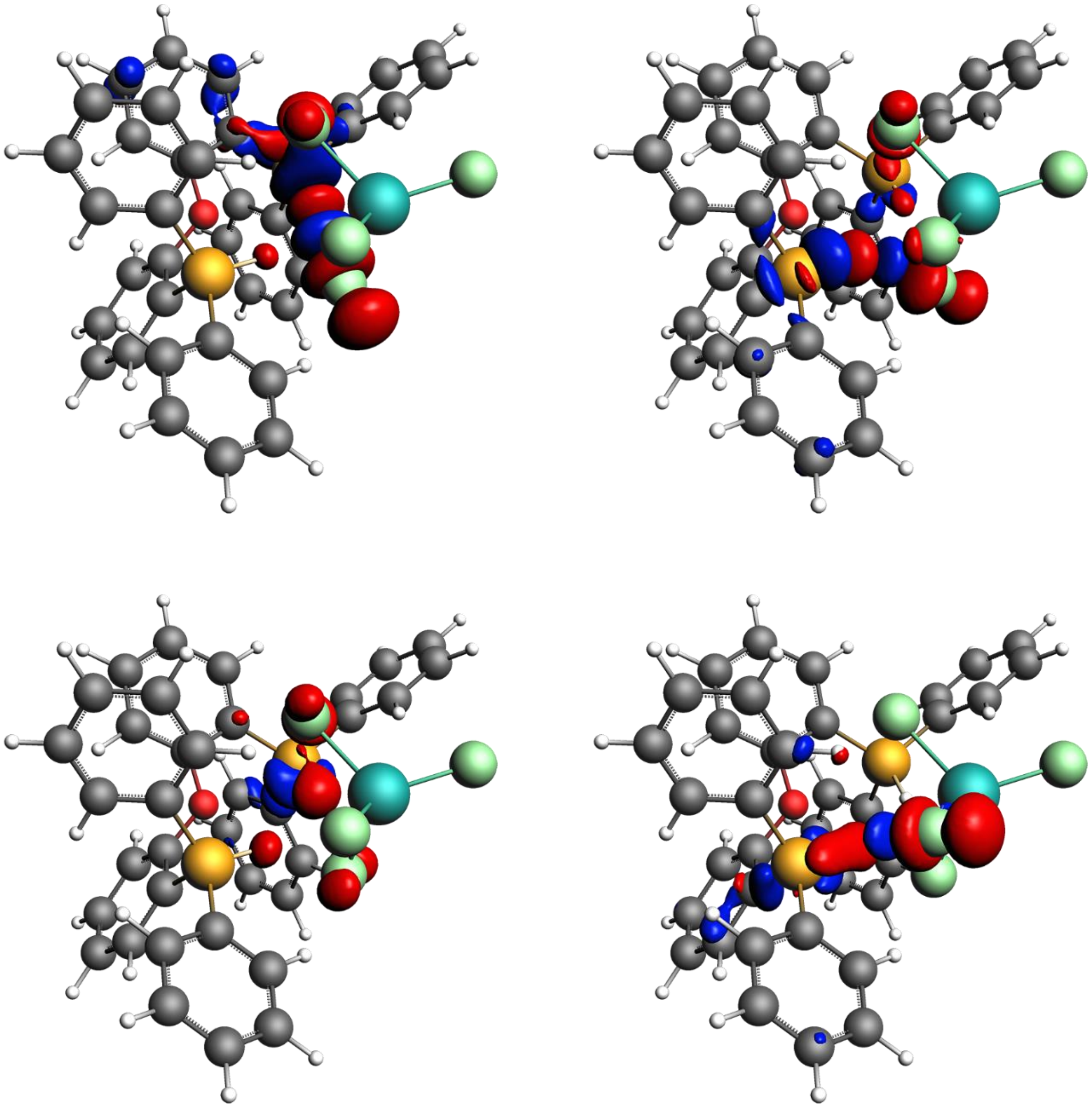

Fig. S11. The calculated deformation density $\Delta\rho_i$ for the $[MnCl_4]^{2-}$ anion with $[H_2DPEphos]^{2+}$ cation (**2**) with NOCV eigenvalues $|V| > 0.1$: left-top – $|V| = 0.1840$; right-top – $|V| = 0.1507$; left-bottom – $|V| = 0.1353$; right-bottom – $|V| = 0.1275$. Red for $\Delta\rho_i < 0$ and blue for $\Delta\rho_i > 0$.

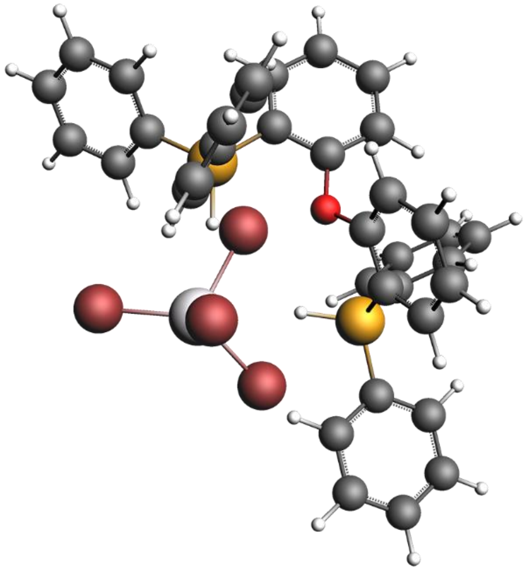

Fig. S12. Optimized structure of the [H_2DPEphos][$ZnBr_4$].

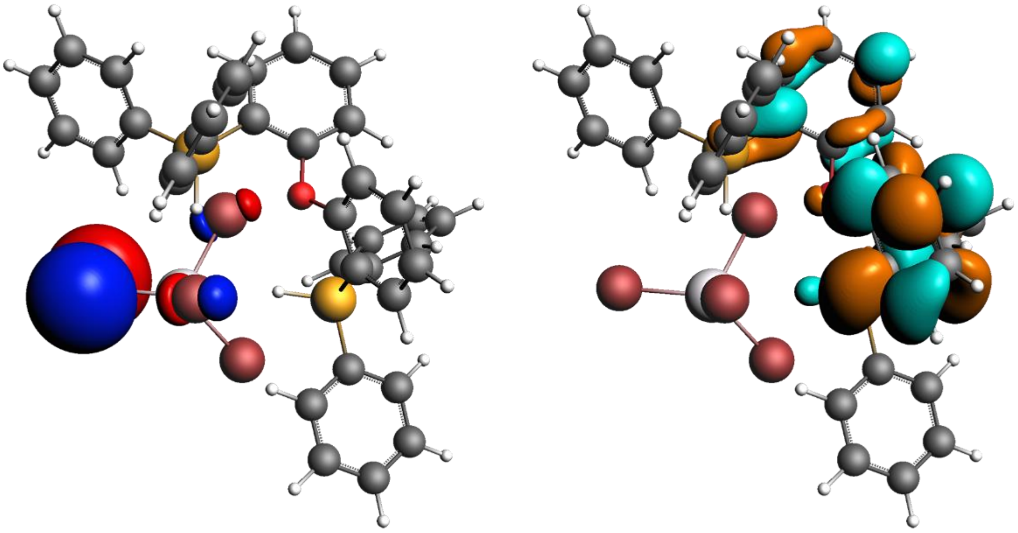

Fig. S13. HOMO (left) and LUMO (right) of the [H_2DPEphos][$ZnBr_4$] using BP86 functional.

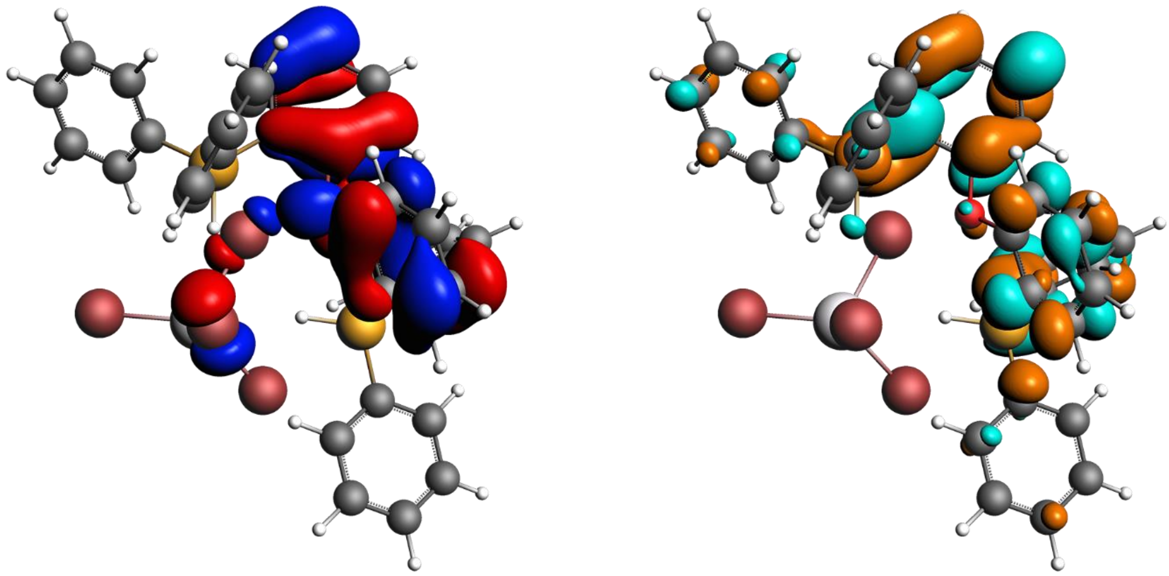

Fig. S14. Occupied (HOMO-11) (left) and unoccupied (LUMO+2) (right) orbitals of the [H_2DPEphos][$ZnBr_4$] involved to the singlet-singlet excitation using BP86 functional.

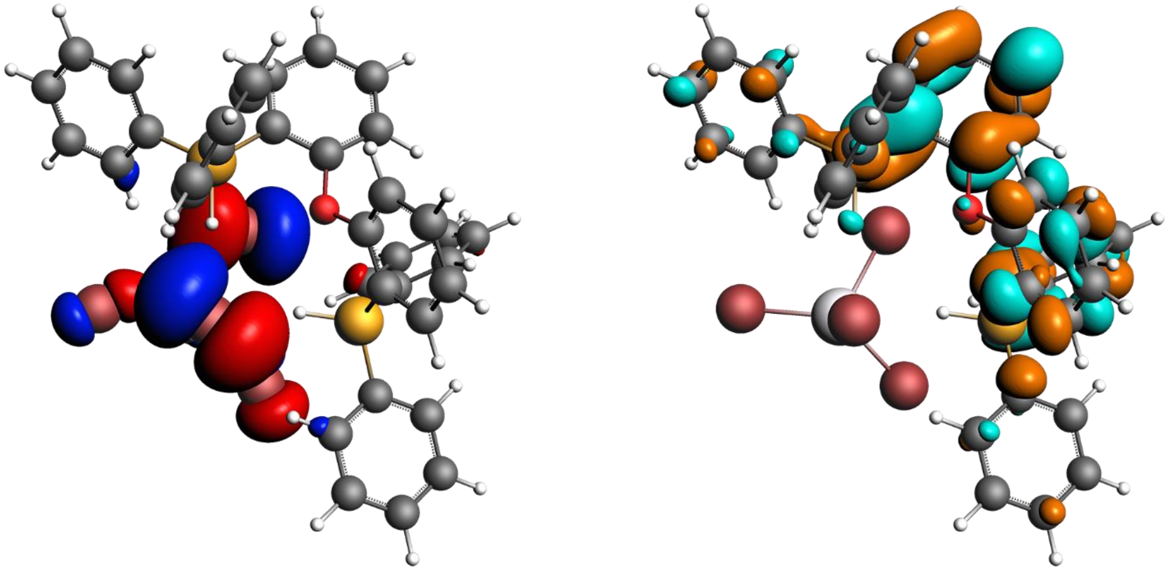

Fig. S15. Occupied (HOMO-7) (left) and unoccupied (LUMO+2) (right) of the [H_2DPEphos][$ZnBr_4$] involved to the singlet-triplet excitation using BP86 functional.

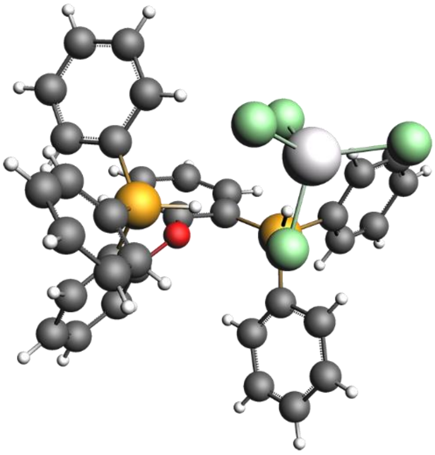

Fig. S16. Optimized structure of the [H_2DPEphos][$ZnCl_4$].

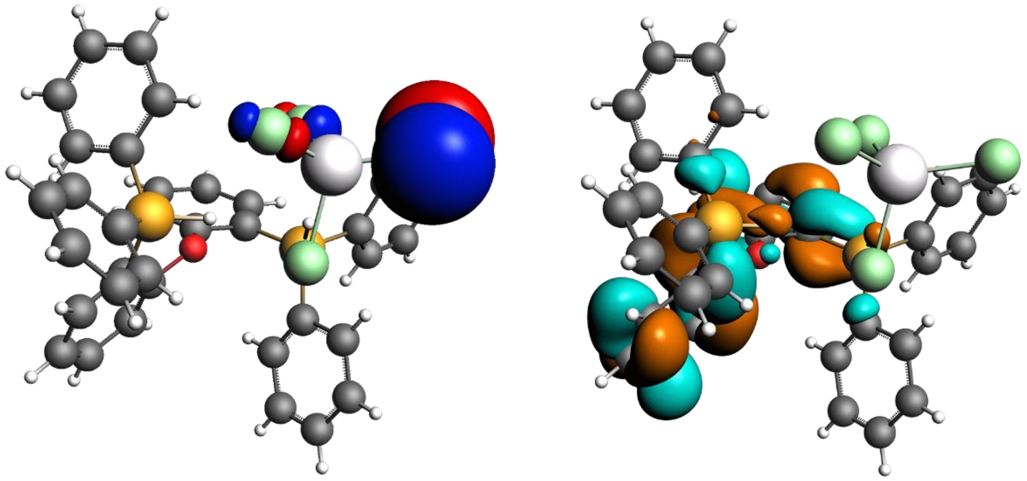

Fig. S17. HOMO (left) and LUMO (right) of the [H_2DPEphos][$ZnCl_4$] using BP86 functional.

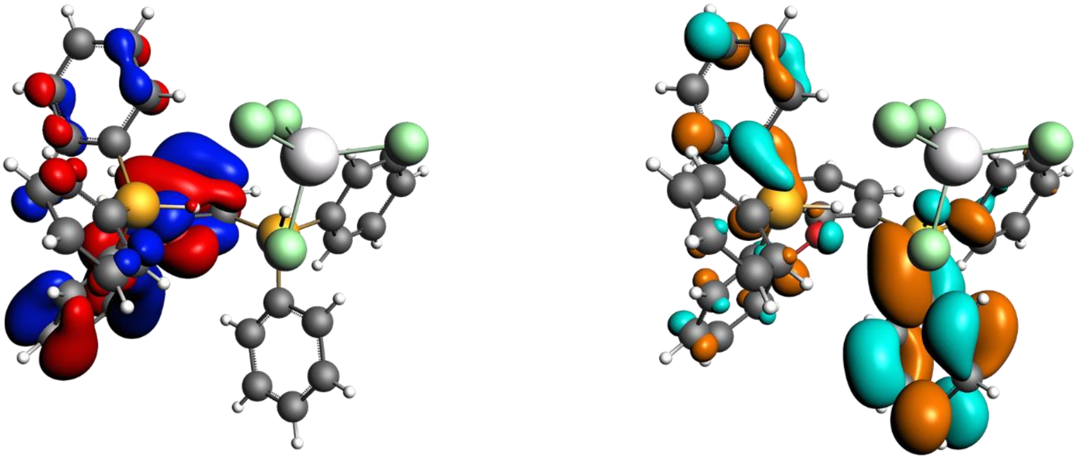

Fig. S18. Occupied (HOMO-11) (left) and unoccupied (LUMO+4) (right) orbitals of the $[H_2DPEphos][ZnCl_4]$ involved to the singlet-singlet excitation using BP86 functional.

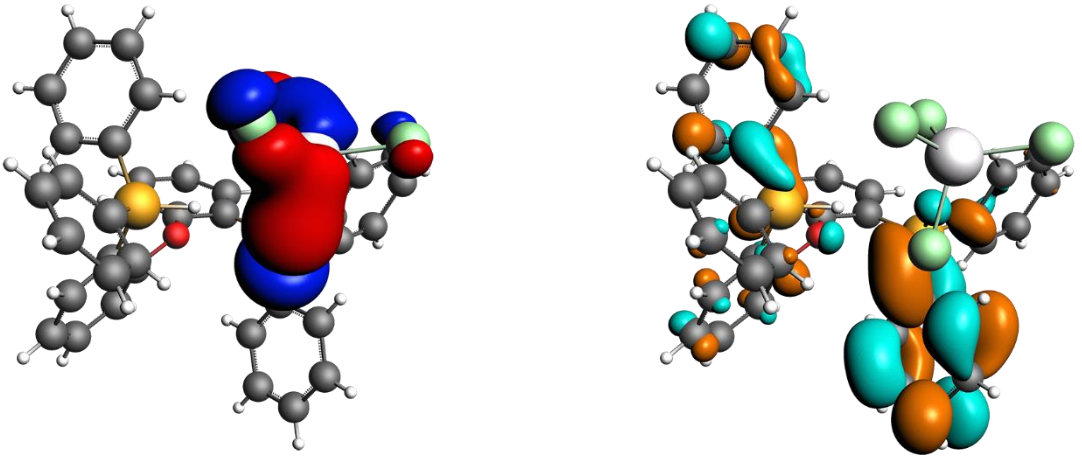

Fig. S19. Occupied (HOMO-10) (left) and unoccupied (LUMO+4) (right) orbitals of the $[H_2DPEphos][ZnCl_4]$ involved to the singlet-triplet excitation using BP86 functional.
